# High foliar K and P resorption efficiencies in old‐growth tropical forests growing on nutrient‐poor soils

**DOI:** 10.1002/ece3.7734

**Published:** 2021-06-14

**Authors:** Ifigenia Urbina, Oriol Grau, Jordi Sardans, Olga Margalef, Guillermo Peguero, Dolores Asensio, Joan LLusià, Romà Ogaya, Albert Gargallo‐Garriga, Leandro Van Langenhove, Lore T. Verryckt, Elodie A. Courtois, Clément Stahl, Jennifer L. Soong, Jerome Chave, Bruno Hérault, Ivan A. Janssens, Emma Sayer, Josep Peñuelas

**Affiliations:** ^1^ CREAF Centre de Recerca Ecològica i Aplicacions Forestals Bellaterra Spain; ^2^ Consejo Superior de Investigaciones Científicas Global Ecology Unit Universidad Autònoma de Barcelona Bellaterra Spain; ^3^ CIRAD UMR EcoFoG (AgroParisTech, CNRS, INRA, Univ Antilles, Univ. Guyane) Kourou French Guiana; ^4^ Department of Biology Centre of Excellence PLECO (Plants and Ecosystems) University of Antwerp Wilrijk Belgium; ^5^ Climate and Ecosystem Science Division Lawrence Berkeley National Laboratory Berkeley CA USA; ^6^ Laboratoire Evolution et Diversité Biologique UMR5174 CNRS–Université Paul Sabatier–IRD Toulouse cedex 9 France; ^7^ Cirad, UR Forêts & Sociétés Université de Montpellier Montpellier France; ^8^ Institut National Polytechnique Félix Houphouët‐Boigny (INP‐HB) Yamoussoukro Ivory Coast; ^9^ Lancaster Environment Centre Lancaster University Lancaster UK; ^10^ Smithsonian Tropical Research Institute Panama Republic of Panama

**Keywords:** nitrogen, nutrient, phosphorus, potassium, resorption, soil, stocks, tropical forest

## Abstract

Resorption is the active withdrawal of nutrients before leaf abscission. This mechanism represents an important strategy to maintain efficient nutrient cycling; however, resorption is poorly characterized in old‐growth tropical forests growing in nutrient‐poor soils. We investigated nutrient resorption from leaves in 39 tree species in two tropical forests on the Guiana Shield, French Guiana, to investigate whether resorption efficiencies varied with soil nutrient, seasonality, and species traits. The stocks of P in leaves, litter, and soil were low at both sites, indicating potential P limitation of the forests. Accordingly, mean resorption efficiencies were higher for P (35.9%) and potassium (K; 44.6%) than for nitrogen (N; 10.3%). K resorption was higher in the wet (70.2%) than in the dry (41.7%) season. P resorption increased slightly with decreasing total soil P; and N and P resorptions were positively related to their foliar concentrations. We conclude that nutrient resorption is a key plant nutrition strategy in these old‐growth tropical forests, that trees with high foliar nutrient concentration reabsorb more nutrient, and that nutrients resorption in leaves, except P, are quite decoupled from nutrients in the soil. Seasonality and biochemical limitation played a role in the resorption of nutrients in leaves, but species‐specific requirements obscured general tendencies at stand and ecosystem level.

## Graphical abstract

Resorption is a key plant nutrition strategy in old‐growth tropical forests. Trees with high foliar nutrient concentration reabsorb more nutrients. Nutrients resorption in leaves, except P, are quite decoupled from nutrients in the soil. Seasonality and biochemical limitation played a role in the resorption of nutrients in leaves, but species‐specific requirements obscured general tendencies at stand and ecosystem level.

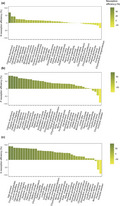

## 1 INTRODUCTION

Tropical forests store large amounts of aboveground biomass, even though they grow on old, highly weathered infertile soils (Gersmehl, 1976; Vitousek & Sanford, 1986). This issue has attracted the attention of the ecological community, and several mechanisms have been proposed to explain this apparent paradox (Bond, 2010; Sayer & Banin, 2016; Turner et al., 2018; Vitousek & Sanford, 1986). One such mechanism is efficient nutrient cycling, whereby greater nutrient retention and internal recycling of essential nutrients by plants minimize nutrient losses in tropical forests with low soil nutrient concentrations (Brant & Chen, 2015; Grau et al., 2017; Vitousek, 1984).

Resorption (also called “retranslocation,” “remobilization,” or “reabsorption”) is the active withdrawal of nutrients before abscission (Fries, 1952; Hill, 1980; Killingbeck, 1996) during senescence, which leads to a series of metabolic changes associated with a decrease in auxin levels, protein breakdown, chlorophyll degradation, and eventually leaf abscission (Fuente & Leopold, 1968). The resorption of foliar nutrients is activated by kinetin signals, which promote nutrient mobilization through the phloem from old senescent leaves (the source) to other plant organs such as stems, roots, or new leaves (sinks) (Hill, 1980). This process of internal nutrient recycling plays an important role in plant nutrition and survival, allowing plants to be more independent of external conditions (Aerts, 1996b; Brant & Chen, 2015; van Heerwaarden et al., 2002; Killingbeck, 1996; Reed et al., 2012). Nutrient resorption can be estimated by resorption efficiency, defined as the percent reduction of a nutrient between green and senesced leaves (Killingbeck, 2003). It can be also estimated as resorption proficiency, defined as the level to which nutrient concentration is reduced in senesced leaves (Aerts, 1996a; Killingbeck, 1996). The resorption of nutrients within a plant can vary greatly from year to year depending on the environmental conditions, such as soil water availability, timing of abscission and the extent of shading (Killingbeck, 2003). In addition, the resorption capacity of plants is determined by biochemical nutrient limitation, whereby the level of nutrient immobilization in leaf compounds (enzymes, proteins, DNA, RNA, etc.) prevents their mobilization from senescing leaves. Overcoming biochemical nutrient limitation can require high energetic costs and some nutrients in structural and recalcitrant compounds, or those used for enzymatic machinery during foliar senescence may be largely unavailable for export from leaves (Killingbeck, 2003).

Nutrient resorption has been postulated as a response of plant species to nutrient‐limiting conditions, which could play an important role in efficient nutrient cycling in tropical forests on infertile soils (Vitousek, 1982). However, despite decades of research, the results about relationship between soil nutrient concentration and nutrient resorption are contradictory and not clear (Aerts & Chapin, 2000; Brant & Chen, 2015; Killingbeck, 1996; Wright & Westoby, 2003; Yuan & Chen, 2009). On another hand, foliar nutrient concentrations seem to play an important role in nutrient resorption. It has been reported at global scale that resorption decreases with increasing foliar nutrient status (Kobe et al., 2005; Vergutz et al., 2012). Several factors can though influence nutrient resorption, which can obscure direct relationships with soil nutrient availability. Water availability is clearly a major driver of resorption, as the reduction in phloem flux transport and water scarcity leads to lower nutrient resorption due to the premature abscission of leaves (Estiarte & Peñuelas, 2015; Killingbeck, 2003). Phenology also substantially affects resorption because flower or fruit production acts as a sink from nutrients in senescent leaves (Estiarte & Peñuelas, 2015; Killingbeck, 2003). Phylogenetic relatedness, understanded as the results of contrasting ecological pressures that make specific niches for different nutrient acquisition strategies, is also expected to play a role in the efficiency of nutrient resorption among plant species, as closely related taxa often have similar resorption efficiencies (Aerts & Van der Piejl, 1993; Killingbeck, 2003; Cantón et al., 2005).

The resorption of nitrogen (N), phosphorus (P), and potassium (K) is of particular interest, because they are three of the most important among the 20 essential nutrients for plant development (Aerts & Chapin, 2000). Nitrogen in plant tissues is mainly present in organic forms as the principal constituent of proteins and nucleic acids. Plants acquire N through root uptake of nitrate or ammonium from the soil and return N to the soil through litterfall. Biological fixation of atmospheric molecular *N* (N_2_) can also provide large inputs of N to support plant growth (Chapin, 1980) that is subsequently cycled within the ecosystem. As the cycling of N is predominantly biological, young natural ecosystems are generally poor in N (Aerts & Chapin, 2000; Turner & Condron, 2013; Walker & Syers, 1976), whereas N is typically not considered to be limiting in old‐growth lowland tropical forests (Tanner et al., 1998; Vitousek & Sanford, 1986).

In contrast to N, P in plant tissues is present in inorganic forms and as a key component of co‐enzymes (ATP and NADPH), nucleic acids, and in larger proportions in phospholipid's membranes. P is provided almost entirely by the weathering of P‐containing minerals (e.g., apatite) from the parent material (Walker & Syers, 1976). Old‐growth tropical forests on ancient soils are therefore generally considered to be P‐limited (Bruijnzeel, 1991; Vitousek et al., 2010) because long‐term weathering of primary minerals depletes total P during soil development and results in increasing dominance of occluded P fractions (Yang & Post, 2011). High concentrations of 1:1 clay and ferric sesquioxides bind P in soils, making it inaccessible to most organisms and bringing the soil system toward what is known as “terminal steady state” (Walker & Syers, 1976).

Potassium represents the most abundant cation (K^+^) in plant cells, and its central role in water economy, photosynthetic capacity, and nutrient transport in plants makes K essential for plant development (Aerts & Chapin, 2000; Sardans & Peñuelas, 2015). Potassium is very soluble and therefore highly mobile in plant tissues and is almost completely provided by the weathering of soil parental material. Old tropical soils, especially those of the Precambrian shield, contain little available K, as described for P, due to the weathering and leaching of this cation from the soil over a very long time (Rosolem et al., 2010; Sayer & Banin, 2016). Potassium has been described as a co‐limiting nutrient for tree growth in wet lowland tropical forests in Costa Rica (Baribault et al., 2012), and the addition of both K and N increased sapling growth in a long‐term fertilization experiment in Panama (Wright et al., 2011).

Based on the differences in the relative mobilities and availabilities of N, P, and K in lowland tropical forests, we would expect the resorption efficiencies to differ among these nutrients. Given that soil N availability is not likely to be limiting and is less mobile in plant tissues due to their highly presence in organic forms, N resorption efficiency should be lower than P or K resorption efficiencies. By contrast, we would expect similar P and K resorption efficiencies because their availability in the soil is low, whereas both elements are highly mobile in plant tissues (Rosolem et al., 2010; Wright et al., 2011). Although previous work demonstrated that resorption efficiency in trees is generally higher for P than N (Vergutz et al., 2012; Yuan & Chen, 2009), less studies have reported K resorption efficiencies (but see Ławniczak, 2011; Vergutz et al., 2012). Furthermore, there is a dearth of information on nutrient resorption for tropical tree species, and thus, the importance of resorption for efficient nutrient cycling in lowland tropical forests remains unquantified. We calculated N, P, and K resorption efficiencies for 39 tropical tree species to investigate the role of phylogeny in determining resorption efficiencies, and to explore potential relationships between nutrient resorption and plant traits. We hypothesized that:

1. Resorption efficiencies for P and K will be higher than for N because of their lower availability in the soil and greater mobility in the plant.
2. Nutrient resorption will be higher for trees with higher foliar nutrient concentrations, as strategy to avoid nutrient loss.
3. Nutrient resorption efficiencies will be higher in the wet than the dry season, because water conductance and nutrient transport are greater in the wet season.
4. Species with slow growth rates and high wood densities (conservative life‐history strategy) will resorb more nutrients than species with high growth rates and low wood densities (acquisitive life‐history strategy), as persistence strategy.
5. Resorption efficiencies will be more similar among more closely related than phylogenetically distant species.

To address these hypotheses, we explored the stocks and the resorption efficiencies of essential elements (N, P, and K) in aboveground (leaves and leaf‐litter) and soil compartments during the wet and dry season in two old‐growth tropical forests growing on old, nutrient‐poor soils.

## 2 MATERIALS AND METHODS

### 2.1 Study area

The study was conducted in French Guiana, a tropical region in the northeastern part of South America (Figure S1a). French Guiana is dominated by tropical forest growing on old, nutrient poor soils developed from the Precambrian Guiana Shield formation (Bongers et al., 2001; Courtois et al., 2018; Epron et al., 2006), including the two study sites: Paracou (5°18′N, 52°53′W) and Nouragues (4°05′N, 52°41′W; Figure S1a). Paracou is located 15 km inland from the coast, in an undisturbed forested area characterized by smooth mosaic hills (Epron et al., 2006; Janssens et al., 1998). The Nouragues Research Station is situated in the center of the country, which is covered by extensive primary forest with granitic hills (Bongers et al., 2001). The climate at both sites is typical of seasonally evergreen tropical rainforests, with a rainy season from December to July and a dry season from August to November. Mean annual rainfall is similar in both study sites (2,990 and 3,041 mm y^‐1^ at Nouragues and Paracou, respectively) (Aguilos et al., 2018; Bongers et al., 2001). The soils at both sites are classified as Oxisols in the USDA soil classification (Anjos et al., 2015), with pH values between 3.7 and 4.5.

### 2.2 Experimental design

At each site, 12 experimental plots were established to represent the spatial variability, including the top of the hills (top plots), the slope of the hills (slope plots), and the bottom of the hills next to rivulets (bottom plots; Figure S1b). At each topographical position, we established four plots of 50 m × 50 m, each with an inner 20 m × 20 m sampling area, and a buffer of 20–100 m between adjacent plots. We marked, tagged, and identified all trees in each plot (50 m × 50 m) and measured diameter at breast height (DBH) for all trees with larger than 10 cm.

Two field campaigns were carried out in 2015, during the rainy season from May to the end of June and during the dry season from the beginning of October until late November. To collect soil samples and leaf litter on the soil surface, we established five sampling points within each plot, one in the center and one in each corner of the inner sampling area (Figure S1c). At each sampling point, we collected all mixed leaf litter from the soil surface within an area of 20 cm × 20 cm and we took soil samples using a soil auger (4‐cm‐diameter) at two depths (0–15 cm and 15–30 cm). The litter samples were oven‐dried at 70°C to constant weight. The soil samples were sieved through a 2‐mm sieve and dried at 105°C for 24 hr to determine dry weight, which was divided by the corer volume to obtain the soil bulk density.

To quantify nutrient resorption, we collected green and senescent leaves from three emergent canopy trees and two subcanopy trees inside the sampling area of each plot. Trees were chosen based on DBH and to maximize the functional traits and number of species sampled (i.e., if two trees belonged to the same species, we chose the next tree of a different species but with similar size). Green leaves (5–10 leaves per tree depending on leaf size) were collected by tree climbers from the upper, mostly sunlit leaves and lower, mainly shaded leaves to represent two contrasting canopy conditions. Green leaves were immediately frozen in liquid N. If the climbers could not reach trees in the 20 m × 20 m sampling area, then we chose trees in the remaining area of the 50 m × 50 m plot following the same criteria. Tree climbers also collected senescent leaves that were still attached to the tree and could be easily detached by shaking the branch. Across both study sites, due to the availability of fresh litter in different seasons and logistical constrains, we were able collected green and senescent leaves from a total of 39 species during the dry season (Table S1) and from a subset of 18 species in the wet season (Table S2). Foliar samples were freeze‐dried (Christ Freeze Dryer ALPHA 1‐2 LDplus, Osterode am Harz, Germany) and senescent leaves were oven‐dried at 70°C to constant weight.

### 2.3 Chemical analysis

Green leaves, litter, senescent leaves, and soil samples were ground with a ball mill (Retsch, model MM400, Restch GmbH) and weighed with an AB204 Mettler Toledo (Mettler Toledo) balance. Foliar C and N were determined by gas chromatography and expressed per unit dry weight. The amount of sample used for subsequent analyses was based on the C and N concentrations (%) of each sample type: For green leaves, senescent leaves, and leaf litter, we used 4.5 mg of pulverized dry sample, and for soil samples, we used 9 mg for 0–15 cm depth (samples with high organic‐matter content) and 11 mg for 15–30 cm depth (samples with moderate to low organic matter content). The C and N concentrations of leaves, senescent leaves, and leaf‐litter samples were analyzed using an elemental analyzer interfaced to an isotope ratio mass spectrometer (PDZ Europa ANCA‐GSL and PDZ Europa 20‐20; Sercon Ltd.), and soils samples were analyzed with an elemental analyzer (Elementar Vario EL Cube or Micro Cube; Elementar Analysensysteme GmbH). During analysis, the samples were interspersed with several replicates of at least two laboratory standards, which were selected to be compositionally similar to the samples being analyzed, and had been previously calibrated against National Institute of Standards and Technology (NIST) Standard Reference Materials (IAEA‐N1, IAEA‐N2, IAEA‐N3, USGS‐40, and USGS‐41).

The concentrations of P and K were determined by inductively coupled plasma (ICP) mass spectrometry (ICP‐MS Agilent 7500 ce) using oven‐dried, pulverized samples (0.1 g soil and 250 mg leaf material) digested in 5 ml of concentrated HNO3 (Milestone Ultrawave digestor; Sorisole, BG, Italy). The accuracies of the digestions and analytical procedures were assessed using blanks (5 ml of HNO_3_ and 2 ml of H_2_O_2_) and certified standards: tomato leaf (NIST 1573a) for biomass and Montana soil (NIST 2711a) and sewage‐amended Soil (CRM005) for soil (NIST).

Soil extractable‐P was determined by two methods, Bray's acid fluoride extraction (Bray‐P; Bray & Kurtz, 1945) and Olsen's bicarbonate extraction (Olsen‐P; Olsen et al., 1954), using sieved, dried soils. P concentrations in both extracts were measured by inductively coupled plasma optical emission spectrometry (iCAP 6300 Duo; Thermo Fisher Scientific, Germany).

To calculate nutrient and carbon stocks per hectare, we used the BRIDGE database (Baraloto et al., 2010) to obtain mean wood density and SLA for all species, and foliar elemental composition (% C, N, P, and K) for trees that were not sampled but were present in our plots. Mean growth rates (mm of DBH increase y^‐1^) were obtained from the Guyafor database (Grau et al., 2017). Preliminary analyses showed that foliar C and nutrient concentrations did not differ significantly between the upper and lower parts of the canopy (Figure S2), so we used the means of both canopy levels for the calculations of stocks and resorption efficiencies.

The total leaf weight for each species was calculated using an allometric coefficient obtained by the power‐law fit, with DBH (cm) as a predictor of leaf weight (kg), using data from Chave et al. (2014). Total leaf weight was then calculated as:

(1) Leafweight(kg)=0.02634434×basalarea

where the basal area of a tree is *π* × (DBH/2)^2^. For more information about the allometric coefficient, see the Supporting Information.

We then calculated foliar C and nutrient stocks per tree as the product of leaf weight and foliar C or nutrient concentrations. Finally, foliar C and nutrient stocks per unit area were calculated as the sum of all the stocks contained in leaves inside the 20 x 20 m sampling area:

(2) $$Y=\sum_{n=1}^{n}X$$

where *Y* represents the stocks in kg/ha of a given element (C, N, P, or K) per plot, *n* represents the number of trees in the 20 m × 20 m sampling area, and *X* represents the stocks of a given element in the leaves of each tree.

Leaf‐litter nutrient and C stocks were estimated as the product of the sample dry weight per unit area and the element concentrations in the sample.

Soil weight per area was calculated as the product of bulk density (g/cm^3^) and core depth (15 cm), and soil C and nutrient stocks were then calculated as the product of soil weight and the C or nutrient concentrations in the sample.

We calculated nutrient‐resorption efficiencies as described by Killingbeck, 1996:

(3) $$\frac{X_{\text{Gl}}‐X_{\text{Sl}}}{X_{\text{Gl}}}\times 100$$

where $X_{\text{Gl}}$ and $X_{\text{Sl}}$ represent the nutrient concentrations of green and senescent leaves, respectively. As we did not measure mass loss during senescence, we were unable to apply the mass‐area loss correction (van Heerwaarden et al., 2002), which could lead to underestimates of resorption efficiencies by *c*. 10% (Han et al., 2013). However, the mass‐loss correction would equally affect the calculated resorption efficiencies of all nutrients. We also calculated resorption proficiencies, defined as nutrient concentration in senescent leaves (Killingbeck, 1996) (Table S4).

### 2.4 Statistical analyses

All analyses were performed in R 3.5.2 (R Core Team, 2017) using the “FactoMineR” package (Le et al., 2008) for multivariate analyses, the “nlme” package (Pinheiro et al., 2017; RCoreTeam, 2017) for linear mixed effects models, and the “picante” (Kembel et al., 2010) and “phytools” (Revell, 2012) packages for phylogenetic analyses.

We used principal component analysis (PCA) with standardized variables to visualize the relationship between the N, P, and K resorption efficiencies, foliar and soil chemical compositions (C, N, P, and K), and functional characteristics (mean growth rate, wood density, DBH, and SLA). We then used linear models to test for differences between sites in C, N, P, or K stocks for each compartment (leaves, leaf litter, upper soil layer, and lower soil layer) separately, in order to determine whether there were differences regarding the nutrient scarcity between the two sites selected.

We used linear mixed effects models to test the influence of season and species on nutrient resorption efficiencies across sites (both studies sites were considered in the models). First, we assessed differences in the resorption efficiencies for N, P, and K using the data for all species sampled in both study sites (*N* = 39), with nutrient as the predictor and species identity as the random effect in the model; the significance of differences between N, P, and K was subsequently determined using Tukey post hoc analysis. We then used the data for the species sampled in both seasons (*N* = 18) to test the effect of season on resorption efficiency for each nutrient separately; models included season as the predictor and species identity as the random effect. Finally, we assessed the influence of tree functional traits (SLA, DBH, wood density, and growth rate) on resorption efficiency; we constructed separate models for each nutrient and trait, using the functional trait as a predictor and species identity as the random effect.

### 2.5 Phylogenetic analysis

We assessed phylogenetic effects on nutrient resorption efficiencies by calculating Pagel's *λ* and Blomberg's *K,* following Münkemüller et al. (2012), that are two of the most common phylogenetic indices used to measure the phylogenetic signals. Both indices quantify the tendency of related species to resemble each other more than species drawn at random from the same phylogenetic tree, where values close to 1 indicate a significant phylogenetic effect. We based our phylogenetic tree on only 31 of the 39 species due to the lack of precise phylogenetic information for eight of the species (*Aniba rosaeodora*, *Chrysophyllum poniferum*, *Eugenia culcullata*, *Inga jenmanii*, *Licania densiflora*, *Myrcia splendens*, *Paloue guianensis,* and *Vochysia sabatieri)*. The phylogenetic tree was based on a phylogenetic tree that covers most tree species present in French Guiana (J. Chave et al., unpublished data).

Results were considered significant at *p* < .05, and we report marginally significant trends for *p* < .1; means are given ±standard deviations throughout.

## 3 RESULTS

Nouragues generally had greater nutrient stocks than Paracou, but P was the scarcest nutrient stored in the leaves, leaf‐litter, and soil in both study sites (Table 1, Figure S3). Soil P stocks were similarly low between sites, whereas soil C, N, and K stocks were higher at Nouragues than Paracou at both depths. Accordingly, soil N:P and K:P ratios (per unit mass) were also higher at Nouragues than Paracou (Figure S4). Leaf litter mass per area was higher in Nouragues than Paracou (Table 1), and leaf litter stocks of N, P, and K were also higher at Nouragues, although C and nutrient stocks in green leaves did not differ significantly between sites (Table 1, Figure S3). The mean foliar N:P ratio (across all species at both study sites) was 29.4 ± 7.0.

**TABLE 1:** Mean values for the mass and C, N, P, and K stocks (kg/ha) at two tropical forest sites in French Guiana, showing stocks in plant compartments (leaves and leaf litter) and in the soil at 0–15 and 15–30 cm depth

Compartment	Site	Mass (kg/ha)	C (kg/ha)	N (kg/ha)	P (kg/ha)	K (kg/ha)
Leaf	Nouragues	8,314 (2,144)	4,112 (2,144)	158 (80.3)	4.65 (2.50)	41.4 (24.2)
Litter	Nouragues	9,544 (1,411)	4,565 (720)	139* (19.2)	2.21 **·** (0.47)	16.4* (5.54)
Soil (0–15 cm)	Nouragues	1,533,250 (275,876)	50,104* (4,436)	3,583* (290)	205 (137)	2,033 (83.6)
Soil (15–30 cm)	Nouragues	1,699,068 (323,762)	28,209* (4,410)	2,222.35* (279)	206 (140)	2,551 (80.4)
Leaf	Paracou	12,243.61 (5,388)	5,689 (5,388)	207 (217)	6.52 (6.41)	54.6 (62.6)
Litter	Paracou	8,641 (1,603)	4,157 (820)	113* (21.6)	1.84 **·** (0.53)	9.68* (6.22)
Soil (0–15 cm)	Paracou	1,675,000 (346,661.1)	36,320* (8,391)	2,566* (630)	158 (61.2)	1,009 **·** (182)
Soil (15–30 cm)	Paracou	1,777,000 (436,931.8)	17,172* (4,552)	1,361* (375)	142 (49.1)	1,265 **·** (108)

Note

Values are means and standard deviations (in parentheses) for *n* = 5 per site for litter and soil, stocks in leaves are the results of allometric equation. Asterisks indicate significant differences at *p* < .05 and dots marginally significant differences at *p* < .1 between sites.

**FIGURE 1:**
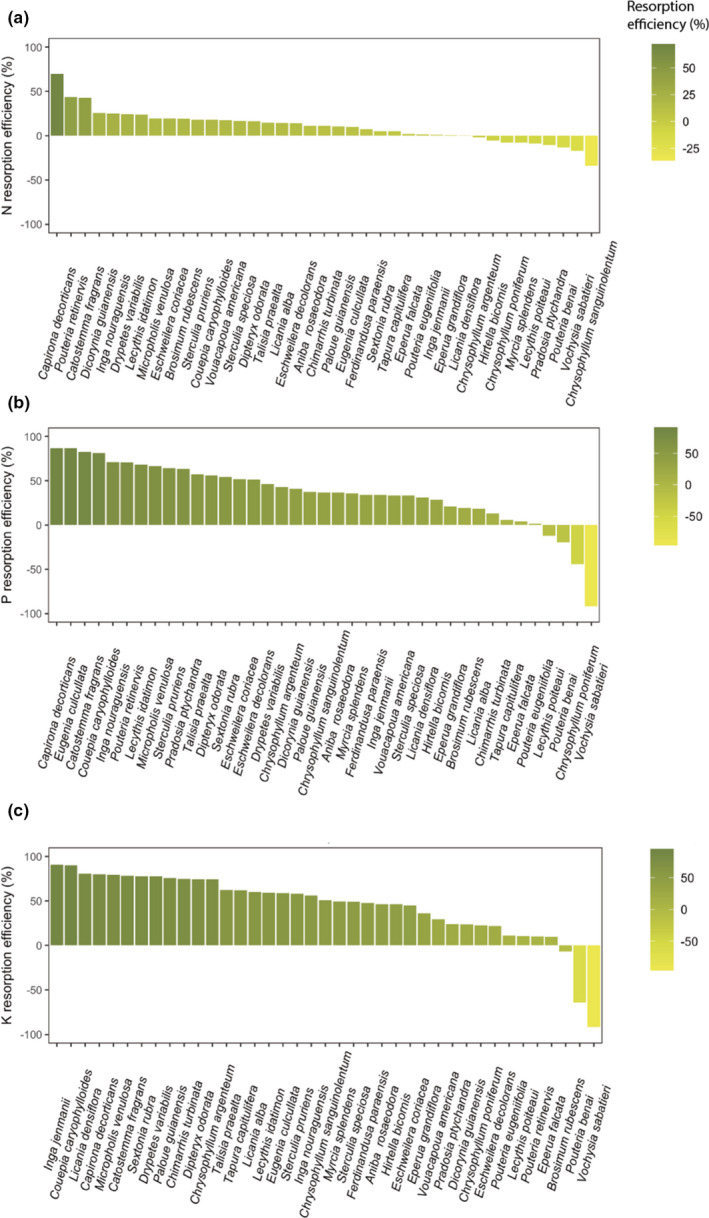
Resorption efficiencies for (a) nitrogen (N), (b) phosphorus (P), and (c) potassium (K) in 39 tropical tree species sampled during the dry season at two lowland tropical forest sites in French Guiana; note that the *y* axis and species rankings differ among panels

**FIGURE 2:**
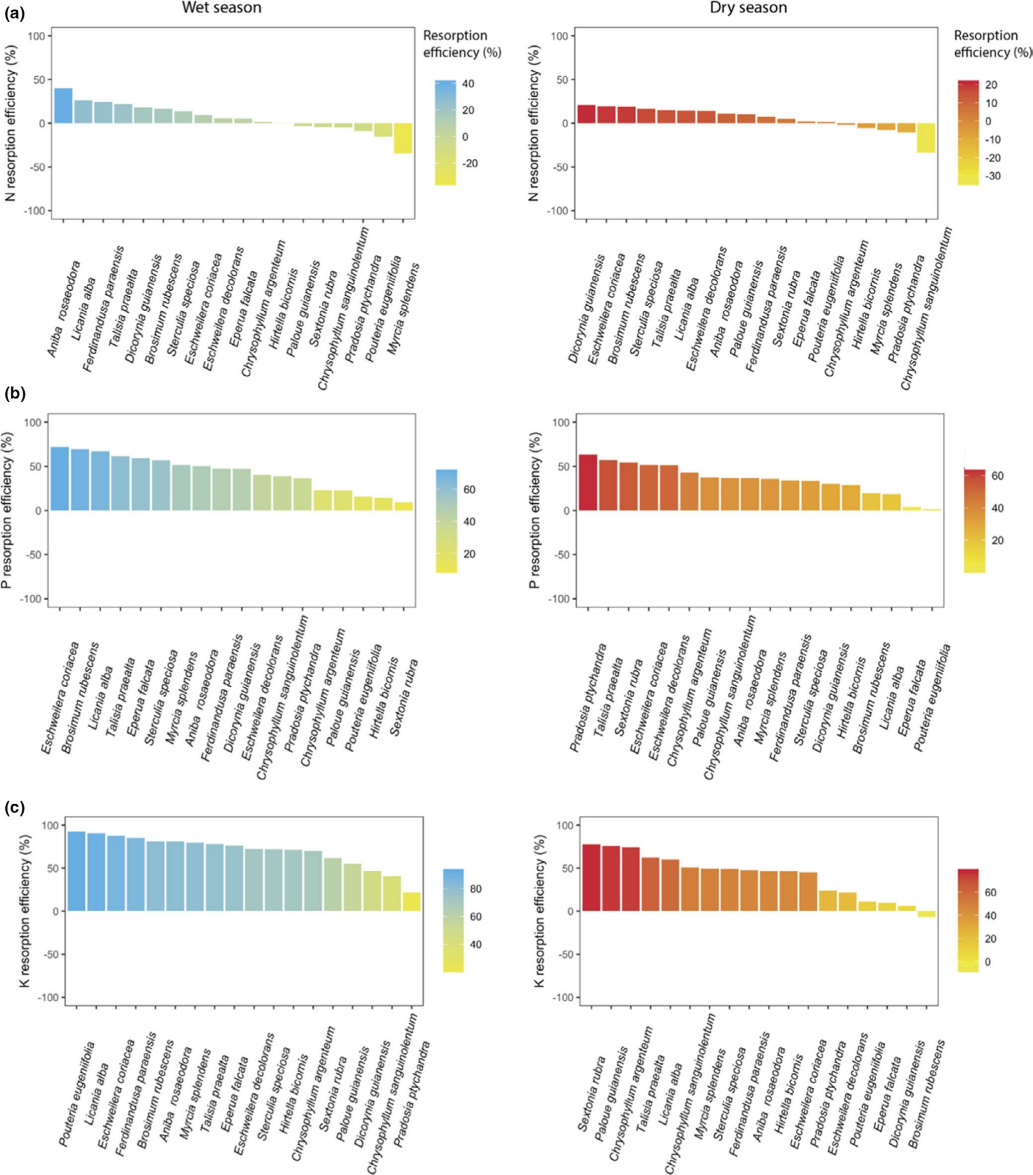
Resorption efficiencies for (a) nitrogen (N), (b) phosphorus (P) and (c) potassium (K) for 18 tropical tree species sampled in the wet (left‐hand panels) and dry (right‐hand panels) seasons at two tropical forest sites in French Guiana; note that the *y* axis and species rankings differ among panels

Resorption efficiencies (mean across all species at both study sites in dry season, *N* = 39) were higher for P and K (35.9% and 44.6%, respectively) than N (10.3%; *p* <.0001. Table 2), and the resorption efficiencies for P and K were generally higher than for N for all species studied (Figure 1), in agreement with our first hypothesis. The range of variation in the resorption efficiencies was smaller for N (−31% to 69.5%) than for P (−91.6% to 86.7%) or K (−91.6% to 90.6%), and there were more negative values for *N* (Figure 1 and Table S1 for resorption efficiencies by species). In contrast, we found lower variation in the P (0.069%–0.007%) and K (0.95%–0.043%) resorption proficiencies than N (2.7%–0.91%) (Table S4), and proficiencies were not related with leaf nutrient concentration. The resorption efficiencies of all three nutrients tended to be higher in the wet than in the dry season (Figure 2). Across all species, K resorption was significantly higher in the wet (70.19 ± 18.7%) than the dry season (41.7 ± 25.26%; *N* = 18. *p* <.01), whereas N and P resorption efficiencies did not differ significantly between seasons (Tables S3), and thus, our third hypothesis was only partially supported.

**TABLE 2:** Significant differences at community level in the N, P, and K resorption efficiencies. (a) Output of the estimated regression parameters, standard errors, and *t* and *p* values for the linear mixed model. The estimated standard deviation associated with the random effect, *σ*_species_, is 23.25. (b) Results of the post hoc test (pairwise comparison) using Tukey's method

(a)					
	Estimate	Standard error	*df*	*t*‐value	*p*‐Value
Intercept	44.61	5.17	76	8.62	<.0001
N	−34.31	5.07	76	−6.75	<.0001
P	−8.71	5.07	76	1.71	.09

(b)					
Contrast	Estimate	Standard error	*df*	*t*‐ratio	*p*‐Value
K‐N	34.31	5.08	76	6.75	<.0001
K‐P	8.71	5.08	76	1.71	.2055
P‐N	−25.6	5.08	76	−5.04	<.0001

**FIGURE 3:**
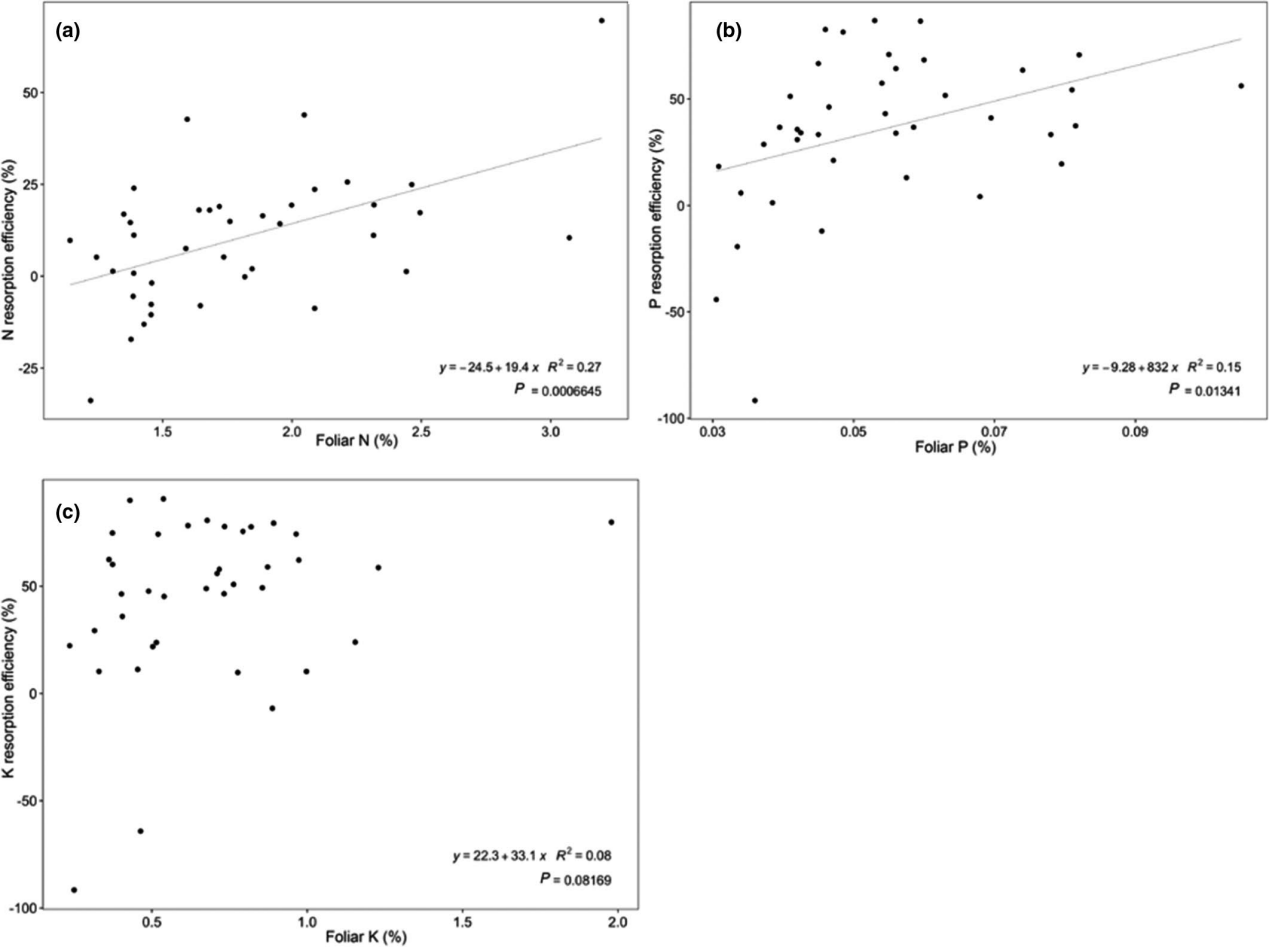
Nutrient resorption efficiencies and their correlations with foliar nutrient concentration (dw/dw) based on the 39 tropical tree species sampled at both study sites in the dry season. (a) N resorption efficiency versus N in leaves, (b) P resorption versus P in leaves, and (c) K resorption versus K in leaves. Coefficients for the significant regressions and *R* ^2^ are displayed in the lower‐right corner of each panel

**FIGURE 4:**
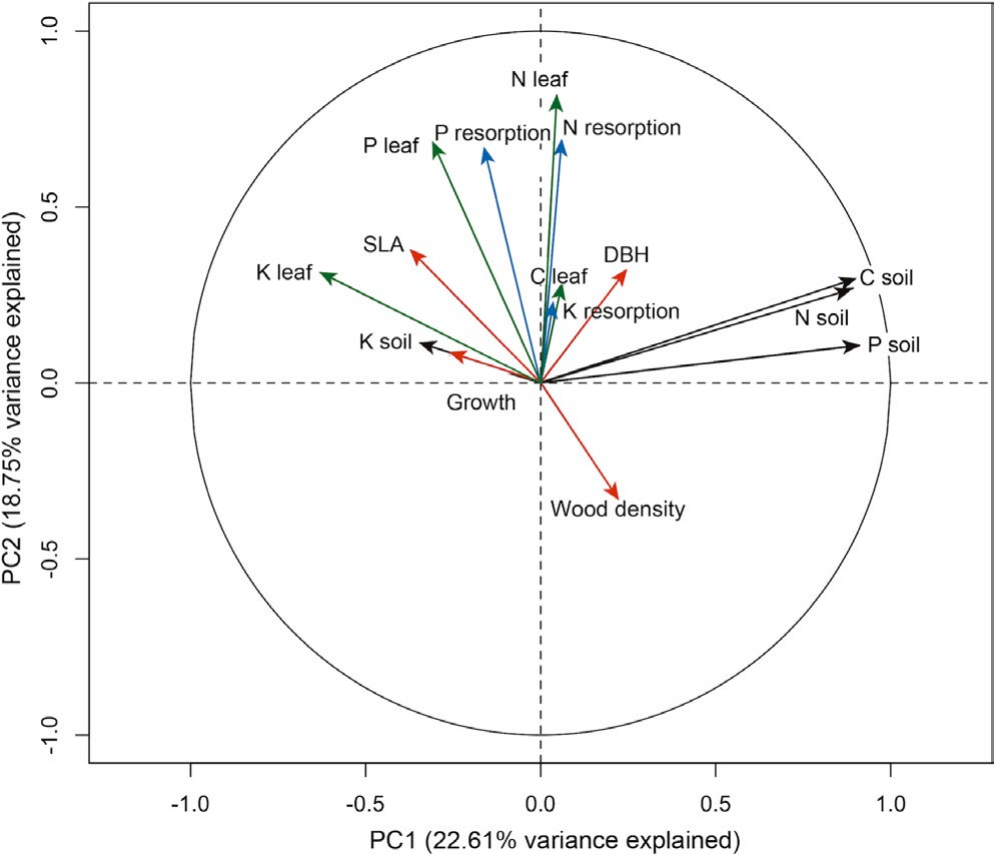
Links between nutrient resorption efficiencies, foliar nutrient concentrations, soil nutrients, and tree species traits visualized in an ordination plot based on principal component analysis (PCA), showing foliar carbon (C), nitrogen (N), phosphorus (P), and potassium (K) concentrations (blue arrows), soil C, N, P, and K concentrations (black arrows), N, P, and K resorption efficiencies (dotted line arrows), as well as tree growth rate, diameter at breast height (DBH), wood density, and specific leaf area (SLA) as functional traits (red arrows)

Nitrogen and P resorption efficiencies increased with foliar N and P concentrations across species (*R* ^2^ = 0.27, *p* < .0001 and *R* ^2^ = 0.15, *p* = .013, respectively), whereas K resorption efficiency was only marginally related to foliar K concentration (*R* ^2^ = 0.08, *p* = .08; Figure 3), so our second hypothesis establishing that nutrient resorption will be higher for trees with higher foliar nutrient concentrations was only partially supported. Phosphorus resorption efficiency declined with increasing total soil P (*R* ^2^ = 0.14, *p* = .02) but was not related to extractable P (Olsen or Bray's P; Figure S5), and there was no relationship between N or K resorption efficiencies and N or K soil concentrations, respectively (Figure S6). Accordingly, the PCA indicated that foliar C, N, and P concentrations were uncoupled from soil C, N and P concentrations. The first and second principal components explained 22.61% and 18.75% of the total variability, respectively, whereby soil variables generally aligned with the first axis, while resorption and leaf concentration aligned with the second axis (Figure 4).

The relationships between nutrient resorption efficiencies and functional characteristics of the species were usually weak but statistically significant (Figure S7). Nitrogen resorption efficiency declined with increasing DBH (*R* ^2^ = 0.14, *p* = .037), whereas P resorption efficiency increased marginally with SLA (*R* ^2^ = 0.13, *p* = .096; Figure S7) and declined with increasing wood density (*R* ^2^ = 0.16, *p* = .026) and there was also a trend toward declining K resorption efficiency with increasing wood density (*R* ^2^ = 0.12, *p* = .052). There was no relationship between nutrient resorption and mean growth rates, and thus, our fourth hypothesis that stated that species with conservative life‐history strategy will resorb more nutrients than species acquisitive life‐history strategy was rejected.

Finally, in contrast to fifth hypothesis, we detected no phylogenetic signal for nutrient resorption efficiency, as Pagel's λ and Blomberg's *K* indices showed no relationship between resorption efficiencies and the phylogenetic distances among species (Table S6).

## 4 DISCUSSION

Our study of 39 tropical tree species across two sites demonstrated that the resorption efficiencies of N, P, and K are partially dependent on foliar nutrient concentrations, but largely decoupled from soil nutrient stocks (except for P), indicating a key role for plant‐internal nutrient cycling for maintaining growth despite infertile tropical soils. Interestingly, despite the lack of a phylogenetic signal for resorption efficiency, we found some evidence that P resorption might be greater in trees with acquisitive life history strategies, contrary to what we expected.

### 4.1 Nutrient stocks

Our results indicated that Nouragues has better nutrient status than Paracou, with higher nutrient inputs from leaf litter to the soil at Nouragues. Nonetheless, the low stocks of P in the soil at both sites (Table 1), as well as high soil N:P and K:P ratios, indicate potential P‐limitation, as expected in this tropical region (Grau et al., 2017; Oliveira et al., 2015; Sayer & Banin, 2016; Vitousek & Farrington, 1997). The N:P ratios we measured (>30) are much higher than the mean value for tropical forests growing on Oxisols (23.5; Townsend et al., 2007), suggesting P limitation (Mo et al., 2019) of plant growth at our study sites.

The allocation patterns of C, N, P, and K stocks were consistent with what has been described for other tropical forests in South America and Africa (Bruijnzeel, 1991; Greenland & Kowal, 1960; Sayer & Banin, 2016; Yavitt et al., 2009), although our study did not include stocks in woody tissue, which represent a large part of the C and nutrients stored in aboveground biomass in tropical forests (Heineman et al., 2016; Tanner, 1985). The higher N stocks in soil than leaves and leaf‐litter conform to our expectation of greater N availability in old tropical soils (Sayer & Banin, 2016; Turner & Condron, 2013; Vitousek et al., 2010). The total concentrations of K in the soil were surprisingly high, and roughly equivalent to N. However, the amount of soil K present in available (in solution or exchangeable) or unavailable (fixed or structural) forms varies by soil type (Rosolem et al., 2010) and our measurements of total soil K may therefore not reflect K availability to plants.

### 4.2 Nutrient resorption and soil nutrient concentration

Plant adaptations to low P availability are common in tropical forests and include a range of strategies such as mycorrhizal associations, carboxylates, and phosphatase production to solubilizes inorganic P bounded in the soil (Batterman et al., 2018; Bucking et al., 2012; Hofmann et al., 2016; Hohenheim & Stuttgart, 1989; Orwin et al., 2011; Sheldrake et al., 2017). However, nutrient resorption from senescent leaves allows plants to be more independent from external resources and saves the metabolic cost of symbiotic associations or the production of enzymes (Brant & Chen, 2015). In support of our first hypothesis, the resorption efficiencies of all the species were generally higher for P and K than for N (Figure 1 and Table S1), suggesting that greater resorption of P and K might be an important mechanism to conserve nutrients that would otherwise be lost through leaching (K) or rendered inaccessible by sorption to soil minerals (P). Indeed, assuming that our mass‐based measurements underestimated resorption by *c*. 10% (Han et al., 2013; Van Heerwaarden et al., 2003), around half of our tree species had P resorption efficiencies above the global average for evergreen angiosperms (50%–60%; Yuan & Chen, 2009; Vergutz et al., 2012) and several species had resorption efficiencies >80% for P and K, which indicates a scarce condition of these nutrients at our sites (Tsujii et al., 2017). By contrast, only one species had an N resorption efficiency >60%, which conforms to our expectation of lower N resorption compared to P or K because of the relatively high availability of N in these old tropical forests (Lambers et al., 2008).

Nutrient resorption efficiency is assumed to be higher when soil nutrient availability is limited (Hidaka & Kitayama, 2011; Reed et al., 2012; Tsujii et al., 2017). Although we found a weak relationship between P resorption efficiencies and total soil P concentrations, there was no relationship between P resorption and extractable P or between N or K resorption efficiencies and soil N or K concentrations. Links between soil fertility and nutrient resorption have been established on a global scale, but the evidence for links between soil fertility and nutrient resorption at regional scales is less clear. Meta‐analyses revealed that phosphorus resorption efficiency increases from high to low latitudes (Yuan & Chen, 2009) and N:P resorption efficiency increases with the latitude and decreases with mean annual temperature and precipitation (Reed et al., 2012). These patterns reflect variation in soil types and nutrient status in different climatic zones (Reed et al., 2012), and greater P resorption efficiency in the tropics is interpreted as a response to low soil P availability (Hidaka & Kitayama, 2011; Yuan & Chen, 2009). However, several empirical studies have found no relationships between nutrient resorption and soil nutrient concentrations along fertility gradients or in fertilization experiments (Aerts, 1996b; Brant & Chen, 2015; Chapin, 1980; Killingbeck, 2003), whereas other studies reported a decrease in N and P resorption efficiencies with fertilization (Mayor et al., 2014; Yuan & Chen, 2015). Total soil P at our study sites was lower than in other tropical forests on similar weathered soils (Yang & Post, 2011; Yavitt, 2000), and the narrow range of soil P concentrations (20–470 ppm) may account for the lack of correlation between P resorption and soil P concentration. However, our study demonstrates clearly that resorption efficiency is higher for P than N, which we interpret as an adaptive strategy of plants in response to low P availability, and the lack of clear relationships between soil fertility and nutrient resorption efficiencies might be explained by biochemical limitation.

### 4.3 Nutrient resorption and biochemical limitation

Biochemical limitation influences resorption efficiency because the level of nutrient immobilization in foliar tissue largely determines the capacity of nutrient recovery from senescent leaves (Killingbeck, 2003; Kobe et al., 2005). Hence, the functional roles and storage forms of nutrients likely also explain the variation in resorption efficiencies. In our study, it is striking that the order of nutrient resorption efficiencies for all species (K ≥ P > N) was the opposite of the level of nutrient immobilization in plant tissues (N > P > K). N is highly immobilized in organic forms in plants (e.g., in proteins, enzymes, and nucleic acids; Chapin, 1980; Cantón et al., 2005), whereas a high proportion of P is present in inorganic forms (Condron et al., 2013; Mo et al., 2019; Walker & Syers, 1976) and K is a free cation in plant cells (Sardans & Peñuelas, 2015). Nutrients more involved in the enzymatic metabolism of foliar senescence, such as N, or energetic forms necessary to export nutrients from leaves, such as P, will be partially unavailable for resorption (Killingbeck, 2003). Potassium can thus be reabsorbed more easily than P, and a greater proportion of P can be reabsorbed than N, which matches the pattern we observed in our study species. In our study, P and K resorption varied more among species than N (Figure 2, Table S1) and greater variation in P than N resorption efficiencies has been observed for different plant growth forms (Aerts & Chapin, 2000) as well as in a global dataset of woody plant species (Han et al., 2013). Greater variability in P versus N resorption efficiency could reflect the comparably greater importance of P resorption for plant nutrient conservation (Han et al., 2013), or the greater extent of N immobilization in plant cell walls (Tsujii et al., 2017). However, foliar concentrations of P are generally more variable than N due to luxury P consumption and greater capacity of plants to store P (Ostertag, 2010); it is therefore possible that variation in resorption efficiencies for N and P is at least partly related to their concentrations in leaves.

In general, trees with higher foliar nutrient concentrations were able to reabsorb more nutrients than trees with low foliar nutrient concentration at our sites, in accordance with our second hypothesis. Although global meta‐analyses demonstrated that nutrient resorption efficiency decreases with increasing leaf nutrient status (Kobe et al., 2005; Vergutz et al., 2012), this relationship may not hold true within highly infertile sites. Several studies in tropical forests on P‐poor soils have demonstrated similarly high P resorption efficiencies or low P concentrations in leaf litter to those measured at our site (e.g., Cai & Bongers, 2007; Richardson et al., 2008; Wood et al., 2011). We thus propose that resorption may be a particularly important plant strategy to avoid nutrient losses in this very infertile tropical soil and high interspecific variation in leaf and litter chemistry (Hättenschwiler et al., 2008) may also entail distinct resorption efficiencies.

### 4.4 Nutrient resorption and the effect of seasonality

In contrast to our third hypothesis, we found no seasonal differences in N and P resorption efficiencies, possibly because higher immobilization of these nutrients in recalcitrant foliar compounds may prevent retranslocation even when phloem water fluxes are high. By contrast, mean K resorption efficiency across all species was higher in the wet than the dry season. Given the high mobility of K, greater resorption efficiencies during the wet season could suggest an important effect of water availability on internal K cycling. However, as K is readily leached from the canopy, greater leaching of K from senesced compared to green leaves during the wet season would present as higher resorption efficiencies, and we cannot currently discount this possibility. Furthermore, the interpretation of seasonal differences in resorption efficiencies may be confounded by differences in the nutrient requirements and acquisition strategies among tree species. The differences in the species rankings (from highest to lowest resorption efficiency) between the wet and dry season (Figure 2) indicates that seasonal differences in resorption may be highly species‐specific.

### 4.5 Effects of phylogeny and species functional characteristics on nutrient resorption

Species with high wood densities are associated with slow growth rates and more conservative strategies (Chave et al., 2009; Muller‐Landau, 2004; Nascimento et al., 2005). As nutrient resorption represents an important plant strategy to conserve nutrients, we hypothesized that more conservative species, with slow growth rates and high wood density, would have higher resorption efficiencies than fast‐growing species. By contrast, we found that species with high wood densities reabsorbed less P and K, and that growth rate was not correlated with N, P or K resorption efficiency. We thus rejected our fourth hypothesis. Furthermore, closely related species did not have more similar resorption efficiencies than distantly related species, and we thus rejected our fifth hypothesis. Previous work in tropical forest established that N and P resorption efficiency declined with increasing leaf toughness, which was attributed to greater allocation of nutrients to structural compounds, which are not easily remobilized (Wood et al., 2011). It is thus conceivable that the allocation of nutrients in species with conservative growth strategies actually reduces their capacity to reabsorb nutrients. Overall, our data did not provide strong evidence for a relationship between resorption efficiency and functional characteristics, although the negative relationship between DBH and N resorption efficiency could reflect the capacity of large trees with extensive roots systems to acquire more N from the soil than smaller trees. Additional work on inter‐ versus intraspecific differences in nutrient resorption and the relationship between functional traits and nutrient resorption could clarify the relative importance of abiotic and biotic controls of resorption efficiencies. The high P and K resorption efficiencies for all species, despite the differences between them and the lack of phylogenetic relationship, suggest that resorption is a highly species‐specific process in these tropical forests to conserve nutrient that are less available in soil.

### 4.6 Conclusions

Our results show that nutrient resorption is an important plant nutrition strategy in these old‐growth tropical forests growing on weathered, nutrient‐poor soils. Trees with high foliar nutrient concentration reabsorb more nutrients, and nutrients that are scarcer in soil presented higher resorption efficiencies. The species‐specific requirements and the functional strategy seem to strongly determine the resorption of nutrients. Furthermore, seasonality and biochemical limitation play also a role in the resorption of nutrients, which make it difficult to obtain a general conclusion about this mechanism in this ecosystem. More studies that address the role of functional strategy and the individual's competition on the nutrient resorption will help to clarify the control of this important nutrient recycling process in tropical forests.

## CONFLICT OF INTEREST

There are not conflicts of interest.

## AUTHOR CONTRIBUTION

**Ifigenia Urbina Barreto:** Conceptualization (lead); Data curation (lead); Formal analysis (lead); Funding acquisition (supporting); Investigation (lead); Methodology (lead); Project administration (lead); Resources (lead); Software (lead); Supervision (lead); Validation (lead); Visualization (lead); Writing‐original draft (lead); Writing‐review & editing (lead). **Oriol Grau:** Conceptualization (lead); Data curation (lead); Formal analysis (lead); Funding acquisition (supporting); Investigation (lead); Methodology (lead); Project administration (supporting); Resources (supporting); Software (supporting); Supervision (lead); Validation (lead); Visualization (lead); Writing‐original draft (equal); Writing‐review & editing (equal). **Jordi Sardans:** Conceptualization (equal); Data curation (supporting); Formal analysis (supporting); Funding acquisition (lead); Investigation (equal); Methodology (supporting); Project administration (lead); Supervision (lead); Validation (equal); Writing‐original draft (equal); Writing‐review & editing (lead). **Olga Margal:** Writing‐review & editing (supporting). **Guille Peguero:** Writing‐review & editing (supporting). **Dolores Asensio:** Writing‐review & editing (supporting). **Juan Llusia:** Writing‐review & editing (supporting). **Romà Ogaya:** Writing‐review & editing (supporting). **Albert Gargallo Gariga:** Writing‐review & editing (supporting). **Leandro Van Langenhove:** Data curation (equal); Formal analysis (supporting); Methodology (equal); Writing‐review & editing (supporting). **Lore Verryckt:** Writing‐review & editing (equal). **Elodie A. Courtois:** Writing‐review & editing (supporting). **Clément Stahl:** Writing‐review & editing (supporting). **Jennifer L. Soong:** Data curation (supporting); Formal analysis (supporting); Investigation (supporting); Methodology (supporting); Supervision (supporting); Validation (supporting); Writing‐review & editing (supporting). **Jerome Chave:** Data curation (lead); Funding acquisition (supporting); Investigation (supporting); Methodology (supporting); Resources (equal); Writing‐review & editing (supporting). **Bruno Hérault:** Data curation (supporting); Formal analysis (supporting); Methodology (supporting); Supervision (supporting); Writing‐review & editing (supporting). **Ivan A. Janssens:** Conceptualization (supporting); Funding acquisition (lead); Investigation (supporting); Resources (supporting); Supervision (supporting); Validation (supporting); Writing‐review & editing (supporting). **Emma Sayer:** Investigation (lead); Supervision (lead); Validation (lead); Visualization (lead); Writing‐review & editing (lead). **Josep Peñuelas:** Conceptualization (lead); Data curation (supporting); Formal analysis (supporting); Funding acquisition (lead); Investigation (lead); Methodology (equal); Project administration (lead); Resources (supporting); Software (supporting); Supervision (lead); Validation (lead); Visualization (lead); Writing‐original draft (lead); Writing‐review & editing (lead).

## Supporting information

Supplementary Material

## Data Availability

The data used in this study will be made publicly available at time of publication in the public repository http://glonuteco.creaf.cat/
