# Supplementary material for: High foliar K and P resorption efficiencies in old‐growth tropical forests growing on nutrient‐poor soils
Source: Ecol Evol. 2021 Jun 14;11(13):8969–82. doi: 10.1002/ece3.7734 (PMC8258221; doi:10.1002/ece3.7734)
Supplement: Supplementary file 1 — Supplementary Material [file ECE3-11-8969-s001.docx]

Supplementary material

Table S1. N, P and K resorption efficiencies (%) for the 39 species sampled in the dry season at both study sites. N show the number of individual sampled and SD in parentheses and not provided for species with only one individual sampled.

| **Species** | ***N*** | **Season** | **N resorption** | | **P resorption** | | **K resorption** | |
| --- | --- | --- | --- | --- | --- | --- | --- | --- |
| *Aniba rosaeodora* Ducke | 1 | Dry | 11.2 |  | 36.7 |  | 46.5 |  |
| *Brosimum rubescens* Taub. | 1 | Dry | 19.0 |  | 19.5 |  | -7.0 |  |
| *Capirona decorticans* Spruce | 1 | Dry | 69.5 |  | 86.8 |  | 79.8 |  |
| *Catostemma fragrans* Benth. | 1 | Dry | 42.7 |  | 82.6 |  | 78.2 |  |
| *Chimarrhis turbinata* DC. | 1 | Dry | 11.1 |  | 13.0 |  | 74.8 |  |
| *Chrysophyllum argenteum* Jacq. | 1 | Dry | -1.9 |  | 43.1 |  | 74.2 |  |
| *Chrysophyllum pomiferum* (Eyma) T.D.Penn. | 1 | Dry | -7.7 |  | -44.3 |  | 22.3 |  |
| *Chrysophyllum sanguinolentum* (Pierre) Baehni | 1 | Dry | -33.8 |  | 36.8 |  | 50.9 |  |
| *Couepia caryophylloides* Benoist | 1 | Dry | 18.0 |  | 81.4 |  | 90.0 |  |
| *Dicorynia guianensis* Amshoff | 5 | Dry | 25.6 | (22.2) | 41.1 | (52.9) | 23.8 | (57.4) |
| *Dipteryx odorata* (Aubl.) Willd. | 1 | Dry | 16.5 |  | 56.2 |  | 74.3 |  |
| *Drypetes variabilis* Uittien | 1 | Dry | 24.0 |  | 46.2 |  | 77.7 |  |
| *Eperua falcata* Aubl. | 8 | Dry | 2.0 | (17.0) | 4.1 | (27.5) | 9.8 | (34.7) |
| *Eperua grandiflora* (Aubl.) Benth | 4 | Dry | 0.8 | (13.6) | 21.1 | (24.6) | 35.9 | (28.1) |
| *Eschweilera coriacea* (DC.) S.A.Mori | 2 | Dry | 19.4 | (3.1) | 51.7 | (12.5) | 45.2 | (21.0) |
| *Eschweilera decolorans* Sandwith | 1 | Dry | 14.2 |  | 51.2 |  | 21.9 |  |
| *Eugenia cucullata* Amshoff | 1 | Dry | 9.7 |  | 86.6 |  | 58.7 |  |
| *Ferdinandusa paraensis* Ducke | 1 | Dry | 7.5 |  | 34.1 |  | 47.7 |  |
| *Hirtella bicornis* Mart. & Zucc. | 2 | Dry | -5.5 | (6.9) | 28.7 | (11.0) | 46.4 | (10.1) |
| *Inga jenmanii* Sandwith | 1 | Dry | 1.3 |  | 33.9 |  | 90.7 |  |
| *Inga nouragensis* Poncy | 1 | Dry | 25.0 |  | 70.9 |  | 55.9 |  |
| *Lecythis idatimon* Aubl. | 1 | Dry | 23.7 |  | 68.3 |  | 59.0 |  |
| *Lecythis poiteaui* O.Berg | 1 | Dry | -8.8 |  | -12.1 |  | 10.3 |  |
| *Licania alba* (Bernoulli) Cuatrec | 7 | Dry | 14.6 | (6.6) | 18.3 | (32.9) | 60.1 | (22.1) |
| *Licania densiflora* Kleinh | 1 | Dry | -0.2 |  | 31.0 |  | 80.7 |  |
| *Micropholis venulosa* (Mart. & Eichler ex Miq.) Pierre | 1 | Dry | 19.4 |  | 66.7 |  | 79.4 |  |
| *Myrcia splendens* (Sw.) DC. | 1 | Dry | -8.0 |  | 35.7 |  | 49.3 |  |
| *Paloue guianensis* Aubl. | 1 | Dry | 10.4 |  | 37.4 |  | 75.5 |  |
| *Pouteria benai* (Aubrév. & Pellegr.)T.D.Penn | 1 | Dry | -13.1 |  | -19.4 |  | -64.2 |  |
| *Pouteria eugeniifolia* (Pierre) Baehni | 2 | Dry | 1.4 | (1.4) | 1.3 | (66.1) | 11.2 | (100.1) |
| *Pouteria retinervis* T.D.Penn. | 1 | Dry | 43.9 |  | 70.7 |  | 10.3 |  |
| *Pradosia ptychandra* (Eyma) T.D.Penn. | 1 | Dry | -10.5 |  | 63.5 |  | 23.9 |  |
| *Sextonia rubra* (Mez) van der Werff | 1 | Dry | 5.2 |  | 54.3 |  | 77.8 |  |
| *Sterculia pruriens* (Aubl.) K.Schum. | 1 | Dry | 18.0 |  | 64.3 |  | 57.9 |  |
| *Sterculia speciosa* K. Schum. | 1 | Dry | 16.9 |  | 33.3 |  | 48.9 |  |
| *Talisia praealta* Radlk | 1 | Dry | 14.9 |  | 57.4 |  | 62.4 |  |
| *Tapura capitulifera* Baill. | 1 | Dry | 5.2 |  | 5.9 |  | 62.2 |  |
| *Vochysia sabatieri* Marc.-Berti | 1 | Dry | -17.2 |  | -91.7 |  | -91.6 |  |
| *Vouacapoua americana* Aubl. | 1 | Dry | 17.3 |  | 33.3 |  | 29.3 |  |

Table S2. N, P and K resorption efficiencies (%) for the 18 species sampled in both the wet and dry seasons. SD in parentheses and not provided for species with only one individual sampled in each season.

| **Species** | ***N*** | **Season** | **N resorption** | | **P resorption** | | **K resorption** | |
| --- | --- | --- | --- | --- | --- | --- | --- | --- |
| *Aniba rosaeodora* Ducke | 1 | Wet | 40.2 |  | 50.0 |  | 81.0 |  |
|  | 1 | Dry | 11.2 |  | 36.7 |  | 46.5 |  |
| *Brosimum rubescens* Taub. | 1 | Wet | 16.8 |  | 69.2 |  | 81.0 |  |
|  | 1 | Dry | 19.0 |  | 19.5 |  | -7.0 |  |
| *Chrysophyllum argenteum* Jacq. | 1 | Wet | 1.4 |  | 23.1 |  | 69.9 |  |
|  | 1 | Dry | -1.9 |  | 43.1 |  | 74.2 |  |
| *Chrysophyllum sanguinolentum* (Pierre) Baehni | 1 | Wet | -5.1 |  | 38.7 |  | 40.7 |  |
|  | 1 | Dry | -33.8 |  | 36.8 |  | 50.9 |  |
| *Dicorynia guianensis* Amshoff | 3 | Wet | 18.0 | (16.0) | 47.0 | (20.49 | 46.6 | (13.9) |
|  | 5 | Dry | 25.6 | (22.2) | 30.0 | (48.7) | 6.1 | (43.3) |
| *Eperua falcata* Aubl. | 3 | Wet | 5.4 | (24.4) | 59.2 | (10.3) | 76.4 | (12.9) |
|  | 8 | Dry | 2.0 | (16.3) | 4.1 | (26.3) | 9.8 | (33.2) |
| *Eschweilera coriacea* (DC.) S.A.Mori | 2 | Wet | 9.4 | (0.2) | 72.0 | (9.1) | 87.7 | (6.9) |
|  | 2 | Dry | 19.4 | (2.5) | 51.7 | (10.2) | 45.2 | (17.2) |
| *Eschweilera decolorans* Sandwith | 1 | Wet | 5.8 |  | 40.4 |  | 72.4 |  |
|  | 1 | Dry | 14.2 |  | 51.2 |  | 21.9 |  |
| *Ferdinandusa paraensis* Ducke | 1 | Wet | 24.3 |  | 47.5 |  | 85.5 |  |
|  | 1 | Dry | 7.5 |  | 34.1 |  | 47.7 |  |
| *Hirtella bicornis* Mart. & Zucc. | 2 | Wet | -0.3 | (0.9) | 14.3 | (21.0) | 71.4 | (18.3) |
|  | 2 | Dry | -5.5 | (5.6) | 28.7 | (9.0) | 46.4 | (8.2) |
| *Licania alba* (Bernoulli) Cuatrec | 5 | Wet | 26.1 | (8.9) | 67.2 | (19.5) | 90.7 | (3.7) |
|  | 7 | Dry | 14.6 | (6.2) | 18.3 | (30.9) | 60.1 | (20.8) |
| *Myrcia splendens* (Sw.) DC. | 1 | Wet | -34.6 |  | 51.7 |  | 79.6 |  |
|  | 1 | Dry | -8.0 |  | 35.7 |  | 49.3 |  |
| *Paloue guianensis* Aubl. | 1 | Wet | -3.1 |  | 22.8 |  | 54.9 |  |
|  | 1 | Dry | 10.4 |  | 37.4 |  | 75.5 |  |
| *Pouteria eugeniifolia* (Pierre) Baehni | 1 | Wet | -15.2 |  | 15.9 |  | 92.2 |  |
|  | 2 | Dry | 1.4 | (1.4) | 1.3 | (66.1) | 11.2 | (100.1) |
| *Pradosia ptychandra* (Eyma) T.D.Penn. | 1 | Wet | -8.8 |  | 36.3 |  | 21.7 |  |
|  | 1 | Dry | -10.5 |  | 63.5 |  | 23.9 |  |
| *Sextonia rubra* (Mez) van der Werff | 1 | Wet | -4.5 |  | 9.5 |  | 61.7 |  |
|  | 1 | Dry | 5.2 |  | 54.3 |  | 77.8 |  |
| *Sterculia speciosa* K. Schum. | 1 | Wet | 13.7 |  | 57.1 |  | 72.1 |  |
|  | 1 | Dry | 16.9 |  | 33.3 |  | 48.9 |  |
| *Talisia praealta* Radlk | 1 | Wet | 22.3 |  | 61.5 |  | 77.9 |  |
|  | 1 | Dry | 14.9 |  | 57.4 |  | 62.4 |  |

Table S3. Significant differences at community level (average of all the species) in the N, P and K resorption efficiencies between different season. Output of the estimated regression parameters, standard errors and *t* and *P* values for the linear mixed models for (a) nitrogen, (b) phosphorus and (c) potassium resorption and the effect of seasonality. The estimated standard deviation associated with the random effect for the model, σ_species_, is 11.88 for nitrogen, 0.002 for phosphorus and 0.02 for potassium.

| *Nitrogen* | Estimate | Standard Error | df | t-value | *P*-value |
| --- | --- | --- | --- | --- | --- |
| Intercept | 6.21 | 3.71 | 17 | 1.67 | 0.11 |
| dry | -0.78 | 3.44 | 17 | -0.22 | 0.82 |

a)

b)

| *Phosphorus* | Estimate | Standard Error | df | t-value | *P*-value |
| --- | --- | --- | --- | --- | --- |
| Intercept | 43.51 | 4.35 | 17 | 9.98 | 0.00 |
| dry | -8.11 | 6.16 | 17 | -1.31 | 0.20 |

| *Potassium* | Estimate | Standard Error | df | t-value | *P*-value |
| --- | --- | --- | --- | --- | --- |
| Intercept | 70.19 | 5.24 | 17 | 13.39 | 0.00 |
| dry | -28.47 | 7.41 | 17 | -3.84 | 0.0013 |

c)

Table S4. Mean and sd values (in parentheses) for N, P and K concentrations in green leaves and senescent leaves (% dw/dw) for the 39 species sampled in the dry season at both study sites. Standard deviation not provided for species with only one individual sampled.

| **Species** | **N (%)** | | | | **P (%)** | | | **K (%)** | | | | |  |
| --- | --- | --- | --- | --- | --- | --- | --- | --- | --- | --- | --- | --- | --- |
|  | leaf | | senescent  leaf | | leaf | | senescent  leaf | | | leaf | | senescent  leaf | |
| *Aniba rosaeodora* Ducke | 1.389 |  | 1.234 |  | 0.040 |  | 0.025 | |  | 0.733 |  | 0.392 |  |
| *Brosimum rubescens* Taub. | 1.720 |  | 1.393 |  | 0.080 |  | 0.064 | |  | 0.888 |  | 0.950 |  |
| *Capirona decorticans* Spruce | 3.197 |  | 0.974 |  | 0.053 |  | 0.007 | |  | 1.979 |  | 0.399 |  |
| *Catostemma fragrans* Benth. | 1.595 |  | 0.914 |  | 0.046 |  | 0.008 | |  | 0.616 |  | 0.134 |  |
| *Chimarrhis turbinata* DC. | 2.315 |  | 2.058 |  | 0.058 |  | 0.050 | |  | 0.373 |  | 0.094 |  |
| *Chrysophyllum argenteum* Jacq. | 1.458 |  | 1.485 |  | 0.055 |  | 0.031 | |  | 0.520 |  | 0.134 |  |
| *Chrysophyllum pomiferum* (Eyma) T.D.Penn. | 1.456 |  | 1.568 |  | 0.031 |  | 0.044 | |  | 0.236 |  | 0.183 |  |
| *Chrysophyllum sanguinolentum* (Pierre) Baehni | 1.221 |  | 1.634 |  | 0.059 |  | 0.037 | |  | 0.763 |  | 0.375 |  |
| *Couepia caryophylloides* Benoist | 1.682 |  | 1.379 |  | 0.049 |  | 0.009 | |  | 0.430 |  | 0.043 |  |
| *Dicorynia guianensis* Amshoff | 2.214 | (0.22) | 1.614 | (0.361) | 0.070 | (0.012) | 0.038 | | (0.033) | 0.514 | (0.134) | 0.364 | (0.236) |
| *Dipteryx odorata* (Aubl.) Willd. | 1.887 |  | 1.576 |  | 0.105 |  | 0.046 | |  | 0.965 |  | 0.248 |  |
| *Drypetes variabilis* Uittien | 1.389 |  | 1.056 |  | 0.047 |  | 0.025 | |  | 0.820 |  | 0.183 |  |
| *Eperua falcata* Aubl. | 1.846 | (0.303) | 1.782 | (0.257) | 0.068 | (0.010) | 0.065 | | (0.021) | 0.776 | (0.275) | 0.669 | (0.279) |
| *Eperua grandiflora* (Aubl.) Benth | 1.388 | (0.036) | 1.379 | (0.198) | 0.047 | (0.006) | 0.036 | | (0.006) | 0.405 | (0.080) | 0.255 | (0.123) |
| *Eschweilera coriacea* (DC.) S.A.Mori | 1.998 | (0.031) | 1.612 | (0.086) | 0.063 | (0.014) | 0.030 | | (0.001) | 0.539 | (0.139) | 0.310 | (0.190) |
| *Eschweilera decolorans* Sandwith | 1.953 |  | 1.675 |  | 0.041 |  | 0.020 | |  | 0.503 |  | 0.393 |  |
| *Eugenia cucullata* Amshoff | 1.142 |  | 1.031 |  | 0.060 |  | 0.008 | |  | 1.229 |  | 0.508 |  |
| *Ferdinandusa paraensis* Ducke | 1.590 |  | 1.470 |  | 0.043 |  | 0.028 | |  | 0.490 |  | 0.256 |  |
| *Hirtella bicornis* Mart. & Zucc. | 1.386 | (0.031) | 1.461 | (0.062) | 0.037 | (0.008) | 0.027 | | (0.010) | 0.402 | (0.216) | 0.227 | (0.156) |
| *Inga jenmanii* Sandwith | 2.442 |  | 2.411 |  | 0.056 |  | 0.037 | |  | 0.537 |  | 0.050 |  |
| *Inga nouragensis* Poncy | 2.463 |  | 1.848 |  | 0.055 |  | 0.016 | |  | 0.710 |  | 0.313 |  |
| *Lecythis idatimon* Aubl. | 2.087 |  | 1.593 |  | 0.060 |  | 0.019 | |  | 0.873 |  | 0.358 |  |
| *Lecythis poiteaui* O.Berg | 2.087 |  | 2.270 |  | 0.046 |  | 0.051 | |  | 0.330 |  | 0.296 |  |
| *Licania alba* (Bernoulli) Cuatrec | 1.375 | (0.097) | 1.175 | (0.132) | 0.031 | (0.004) | 0.024 | | (0.008) | 0.373 | (0.156) | 0.142 | (0.085) |
| *Licania densiflora* Kleinh | 1.818 |  | 1.821 |  | 0.042 |  | 0.029 | |  | 0.678 |  | 0.131 |  |
| *Micropholis venulosa* (Mart. & Eichler ex Miq.) Pierre | 2.317 |  | 1.867 |  | 0.045 |  | 0.015 | |  | 0.892 |  | 0.184 |  |
| *Myrcia splendens* (Sw.) DC. | 1.646 |  | 1.778 |  | 0.042 |  | 0.027 | |  | 0.856 |  | 0.434 |  |
| *Paloue guianensis* Aubl. | 3.072 |  | 2.751 |  | 0.082 |  | 0.051 | |  | 0.793 |  | 0.194 |  |
| *Pouteria benai* (Aubrév. & Pellegr.)T.D.Penn | 1.428 |  | 1.614 |  | 0.034 |  | 0.040 | |  | 0.464 |  | 0.761 |  |
| *Pouteria eugeniifolia* (Pierre) Baehni | 1.307 | (0.025) | 1.290 | (0.043) | 0.039 |  | 0.038 | | (0.025) | 0.454 | (0.048) | 0.379 | (0.412) |
| *Pouteria retinervis* T.D.Penn. | 2.048 |  | 1.149 |  | 0.082 |  | 0.024 | |  | 0.998 |  | 0.895 |  |
| *Pradosia ptychandra* (Eyma) T.D.Penn. | 1.456 |  | 1.608 |  | 0.074 |  | 0.027 | |  | 1.155 |  | 0.878 |  |
| *Sextonia rubra* (Mez) van der Werff | 1.737 |  | 1.646 |  | 0.081 |  | 0.037 | |  | 0.734 |  | 0.163 |  |
| *Sterculia pruriens* (Aubl.) K.Schum. | 1.641 |  | 1.345 |  | 0.056 |  | 0.020 | |  | 0.717 |  | 0.302 |  |
| *Sterculia speciosa* K. Schum. | 1.350 |  | 1.122 |  | 0.045 |  | 0.030 | |  | 0.675 |  | 0.345 |  |
| *Talisia praealta* Radlk | 1.760 |  | 1.498 |  | 0.054 |  | 0.023 | |  | 0.362 |  | 0.136 |  |
| *Tapura capitulifera* Baill. | 1.245 |  | 1.180 |  | 0.034 |  | 0.032 | |  | 0.973 |  | 0.368 |  |
| *Vochysia sabatieri* Marc.-Berti | 1.378 |  | 1.614 |  | 0.036 |  | 0.069 | |  | 0.250 |  | 0.479 |  |
| *Vouacapoua americana* Aubl. | 2.495 |  | 2.063 |  | 0.078 |  | 0.052 | |  | 0.316 |  | 0.223 |  |

Table S5. Mean and SD values (in parentheses) for C, N, P and K concentrations (% dw/dw) in soil by plot in each study sites.

| **Site** | **Plot** | **Depth (cm)** | **C (%)** | | **N (%)** | | **P (%)** | | **K (%)** | |
| --- | --- | --- | --- | --- | --- | --- | --- | --- | --- | --- |
| Paracou | top | 0-15 | 1.997 | (0.485) | 0.141 | (0.030) | 0.008 | (0.003) | 0.059 | (0.025) |
| Paracou | top | 15-30 | 0.789 | (0.189) | 0.062 | (0.013) | 0.007 | (0.003) | 0.083 | (0.070) |
| Paracou | slope | 0-15 | 2.480 | (0.819) | 0.171 | (0.046) | 0.013 | (0.004) | 0.074 | (0.046) |
| Paracou | slope | 15-30 | 1.330 | (0.466) | 0.102 | (0.033) | 0.012 | (0.004) | 0.091 | (0.076) |
| Paracou | bottom | 0-15 | 2.102 | (0.704) | 0.151 | (0.047) | 0.008 | (0.003) | 0.055 | (0.026) |
| Paracou | bottom | 15-30 | 0.927 | (0.492) | 0.075 | (0.029) | 0.007 | (0.004) | 0.055 | (0.013) |
| Nouragues | top | 0-15 | 4.331 | (0.920) | 0.301 | (0.048) | 0.031 | (0.009) | 0.041 | (0.010) |
| Nouragues | top | 15-30 | 2.386 | (0.584) | 0.181 | (0.038) | 0.029 | (0.009) | 0.039 | (0.008) |
| Nouragues | slope | 0-15 | 3.083 | (0.788) | 0.220 | (0.046) | 0.009 | (0.002) | 0.123 | (0.107) |
| Nouragues | slope | 15-30 | 1.555 | (0.379) | 0.123 | (0.024) | 0.008 | (0.001) | 0.130 | (0.119) |
| Nouragues | bottom | 0-15 | 2.804 | (1.026) | 0.206 | (0.069) | 0.006 | (0.002) | 0.210 | (0.177) |
| Nouragues | bottom | 15-30 | 1.269 | (0.615) | 0.104 | (0.041) | 0.005 | (0.001) | 0.244 | (0.202) |

Table S6 (a) Results for Pagel’s and Blomberg’s indices used to calculate the phylogenetic signals (value and significance) based on 31 species. (b) Phylogenetic tree based on the 31 species. *Aniba rosaeodora, Chrysophyllum poniferum, Eugenia culcullata, Inga jenmanii,* *Licania densiflora*, *Myrcia splendens*, *Paloue guianensis*, *Vochysia sabatieri* could not be included in the phylogenetic analysis due to the lack of genetic sequences.

|  | **N resorption** | | **P resorption** | | **K resorption** | |
| --- | --- | --- | --- | --- | --- | --- |
| Phylogenetic signal |  | *P* |  | *P* |  | *P* |
| Pagel’s λ | 6.64 × 10^-5^ | 1 | 6.64 × 10^-5^ | 1 | 6.64 × 10^-5^ | 1 |
| Blomberg’s *K* | 0.128 | 0.393 | 0.119 | 0.374 | 0.119 | 0.373 |

a)


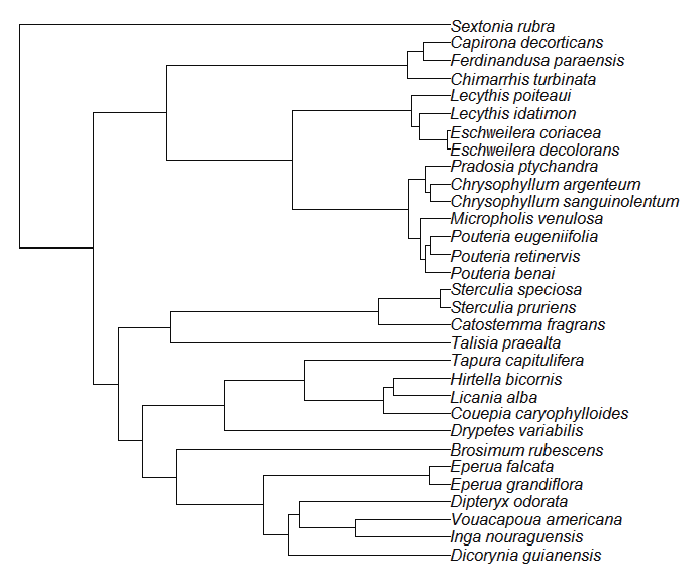


b)

Table S7. N, P and K resorption proficiencies (%) (nutrient concentration in senescent leaves) for the 39 species sampled in the dry season at both study sites. N show the number of individual sampled and SD in parentheses and not provided for species with only one individual sampled.

| ***Species*** | ***N*** | **Season** | **N proficiency** | | **P proficiency** | | **K proficiency** | |
| --- | --- | --- | --- | --- | --- | --- | --- | --- |
| *Aniba rosaeodora* Ducke | 1 | Dry | 1.234 |  | 0.025 |  | 0.392 |  |
| *Brosimum rubescens* Taub. | 1 | Dry | 1.393 |  | 0.064 |  | 0.950 |  |
| *Capirona decorticans* Spruce | 1 | Dry | 0.974 |  | 0.007 |  | 0.399 |  |
| *Catostemma fragrans* Benth. | 1 | Dry | 0.914 |  | 0.008 |  | 0.134 |  |
| *Chimarrhis turbinata* DC. | 1 | Dry | 2.058 |  | 0.050 |  | 0.094 |  |
| *Chrysophyllum argenteum* Jacq. | 1 | Dry | 1.485 |  | 0.031 |  | 0.134 |  |
| *Chrysophyllum pomiferum* (Eyma) T.D.Penn. | 1 | Dry | 1.568 |  | 0.044 |  | 0.183 |  |
| *Chrysophyllum sanguinolentum* (Pierre) Baehni | 1 | Dry | 1.634 |  | 0.037 |  | 0.375 |  |
| *Couepia caryophylloides* Benoist | 1 | Dry | 1.379 |  | 0.009 |  | 0.043 |  |
| *Dicorynia guianensis* Amshoff | 5 | Dry | 1.614 | (0.361) | 0.038 | (0.033) | 0.364 | (0.236) |
| *Dipteryx odorata* (Aubl.) Willd. | 1 | Dry | 1.576 |  | 0.046 |  | 0.248 |  |
| *Drypetes variabilis* Uittien | 1 | Dry | 1.056 |  | 0.025 |  | 0.183 |  |
| *Eperua falcata* Aubl. | 8 | Dry | 1.782 | (0.257) | 0.065 | (0.021) | 0.669 | (0.279) |
| *Eperua grandiflora* (Aubl.) Benth | 4 | Dry | 1.379 | (0.198) | 0.036 | (0.006) | 0.255 | (0.123) |
| *Eschweilera coriacea* (DC.) S.A.Mori | 2 | Dry | 1.612 | (0.086) | 0.030 | (0.001) | 0.310 | (0.190) |
| *Eschweilera decolorans* Sandwith | 1 | Dry | 1.675 |  | 0.020 |  | 0.393 |  |
| *Eugenia cucullata* Amshoff | 1 | Dry | 1.031 |  | 0.008 |  | 0.508 |  |
| *Ferdinandusa paraensis* Ducke | 1 | Dry | 1.470 |  | 0.028 |  | 0.256 |  |
| *Hirtella bicornis* Mart. & Zucc. | 2 | Dry | 1.461 | (0.062) | 0.027 | (0.010) | 0.227 | (0.156) |
| *Inga jenmanii* Sandwith | 1 | Dry | 2.411 |  | 0.037 |  | 0.050 |  |
| *Inga nouragensis* Poncy | 1 | Dry | 1.848 |  | 0.016 |  | 0.313 |  |
| *Lecythis idatimon* Aubl. | 1 | Dry | 1.593 |  | 0.019 |  | 0.358 |  |
| *Lecythis poiteaui* O.Berg | 1 | Dry | 2.270 |  | 0.051 |  | 0.296 |  |
| *Licania alba* (Bernoulli) Cuatrec | 7 | Dry | 1.175 | (0.132) | 0.024 | (0.008) | 0.142 | (0.085) |
| *Licania densiflora* Kleinh | 1 | Dry | 1.821 |  | 0.029 |  | 0.131 |  |
| *Micropholis venulosa* (Mart. & Eichler ex Miq.) Pierre | 1 | Dry | 1.867 |  | 0.015 |  | 0.184 |  |
| *Myrcia splendens* (Sw.) DC. | 1 | Dry | 1.778 |  | 0.027 |  | 0.434 |  |
| *Paloue guianensis* Aubl. | 1 | Dry | 2.751 |  | 0.051 |  | 0.194 |  |
| *Pouteria benai* (Aubrév. & Pellegr.)T.D.Penn | 1 | Dry | 1.614 |  | 0.040 |  | 0.761 |  |
| *Pouteria eugeniifolia* (Pierre) Baehni | 2 | Dry | 1.290 | (0.043) | 0.038 | (0.025) | 0.379 | (0.412) |
| *Pouteria retinervis* T.D.Penn. | 1 | Dry | 1.149 |  | 0.024 |  | 0.895 |  |
| *Pradosia ptychandra* (Eyma) T.D.Penn. | 1 | Dry | 1.608 |  | 0.027 |  | 0.878 |  |
| *Sextonia rubra* (Mez) van der Werff | 1 | Dry | 1.646 |  | 0.037 |  | 0.163 |  |
| *Sterculia pruriens* (Aubl.) K.Schum. | 1 | Dry | 1.345 |  | 0.020 |  | 0.302 |  |
| *Sterculia speciosa* K. Schum. | 1 | Dry | 1.122 |  | 0.030 |  | 0.345 |  |
| *Talisia praealta* Radlk | 1 | Dry | 1.498 |  | 0.023 |  | 0.136 |  |
| *Tapura capitulifera* Baill. | 1 | Dry | 1.180 |  | 0.032 |  | 0.368 |  |
| *Vochysia sabatieri* Marc.-Berti | 1 | Dry | 1.614 |  | 0.069 |  | 0.479 |  |
| *Vouacapoua americana* Aubl. | 1 | Dry | 2.063 |  | 0.052 |  | 0.223 |  |

Figure S1. Location of the study sites and experimental design. (a) Location of French Guiana in South America and the two study sites: the Paracou and Nouragues research stations (red dots). (b) Topographic maps of Paracou (top panel) and Nouragues (lower panel). Twelve plots four for each topographic level (in the figure, B = bottom of the hill (blue), S = slope plots in the middle of the hills (yellow) and T = top plots in the top of the hill (red), were established at each site to catch spatial variability. (c) Experimental plots (50 × 50 m), buffer zone and the sampling area (20 × 20 m) (solid line). Circles in the sampling area indicate the sampling points for the soil and leaf litter.


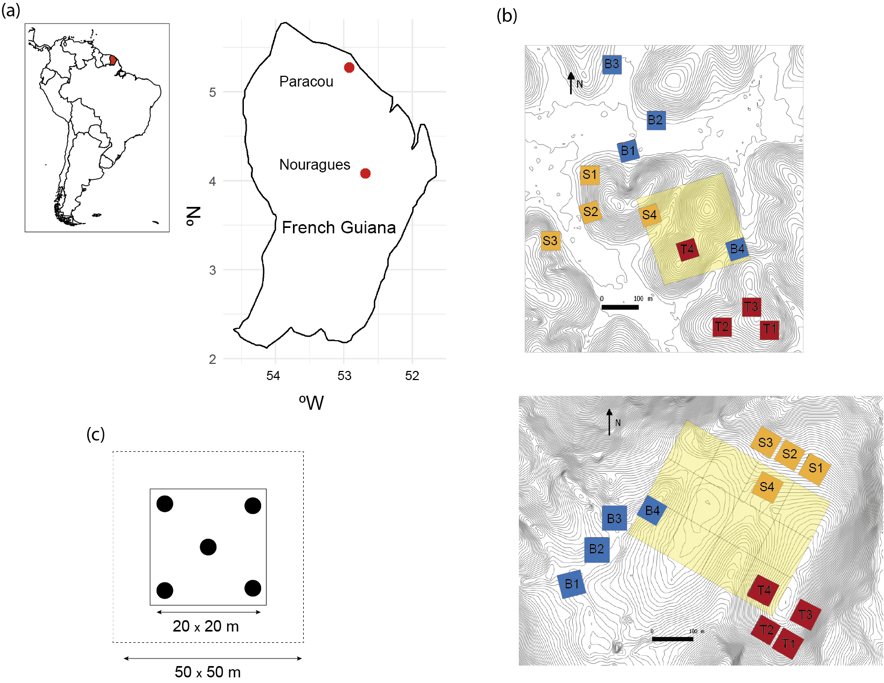


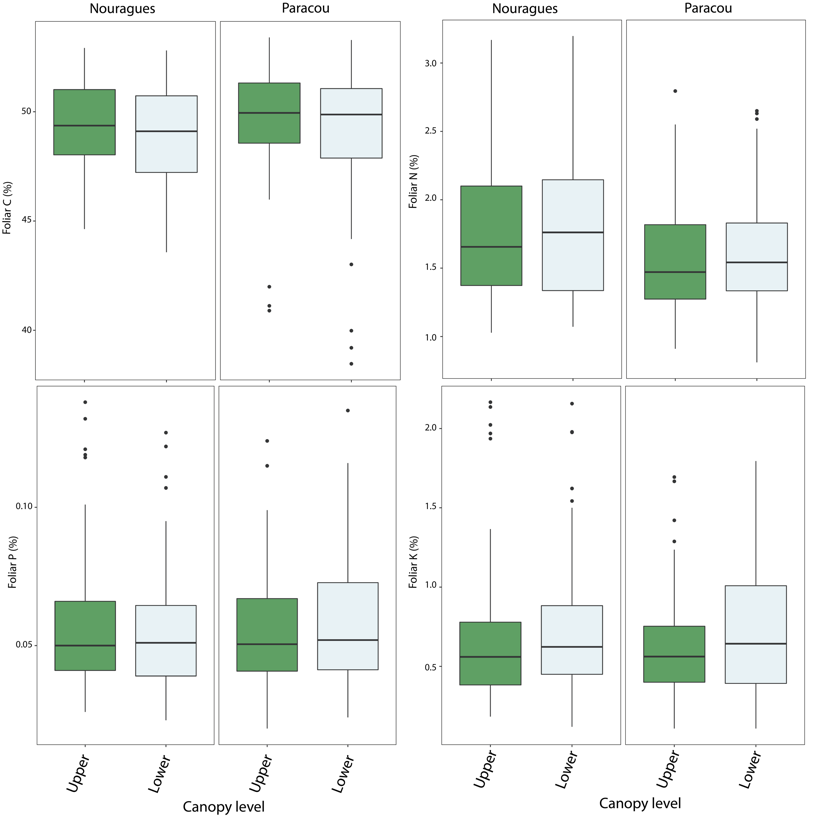
Figure S2. C, N, P and K concentrations (dw/dw) in the upper and lower tree canopy for Nouragues (left panels) and Paracou (right panels). Boxes represent the second and third quartiles, the black lines inside the boxes represent medians, bars represent the first (top) and fourth (down) quartiles, and dots represent outliers.

Figure S3. Nutrient stocks in leaves, leaf-litter and soil (kg ha^-1^) at two lowland tropical forest sites in French Guiana: Nouragues (left-hand panels) and Paracou (right-hand panels), showing (a) carbon (C), (b) nitrogen (N), (c) phosphorus (P) and (d) potassium (K). Error bars indicate standard errors, asterisks indicate significant differences at p < 0.05 and dots marginally differences at p <0.1 between sites.


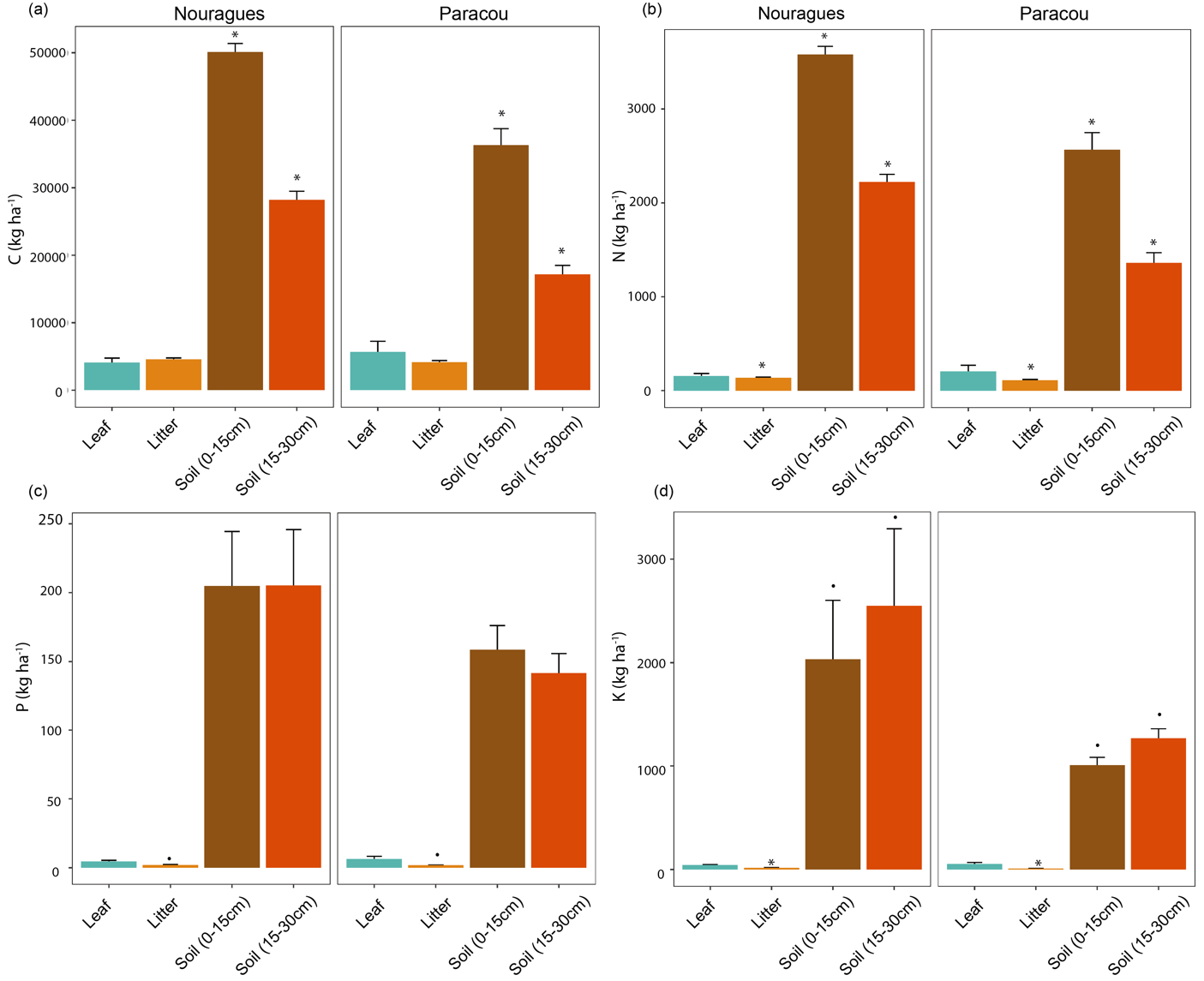


Figure S4. N:P (upper panels) and K:P (lower panels) ratios for the 0-15 and 15-30 cm soil layers at the two sites, Nouragues (left panels) and Paracou (right panels). ﻿ Boxes represent the second and third quartiles, the black lines inside the boxes represent medians, bars represent the first (top) and fourth (down) quartiles, and dots represent outliers.


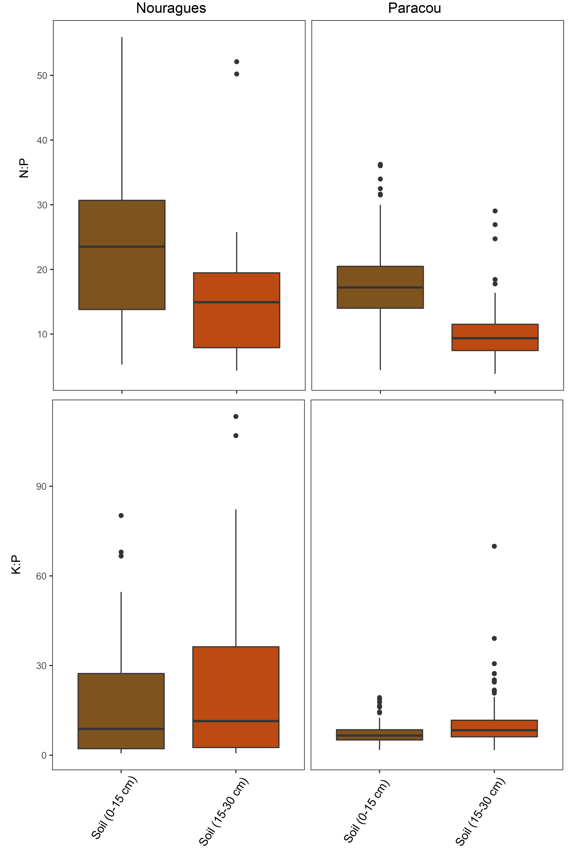


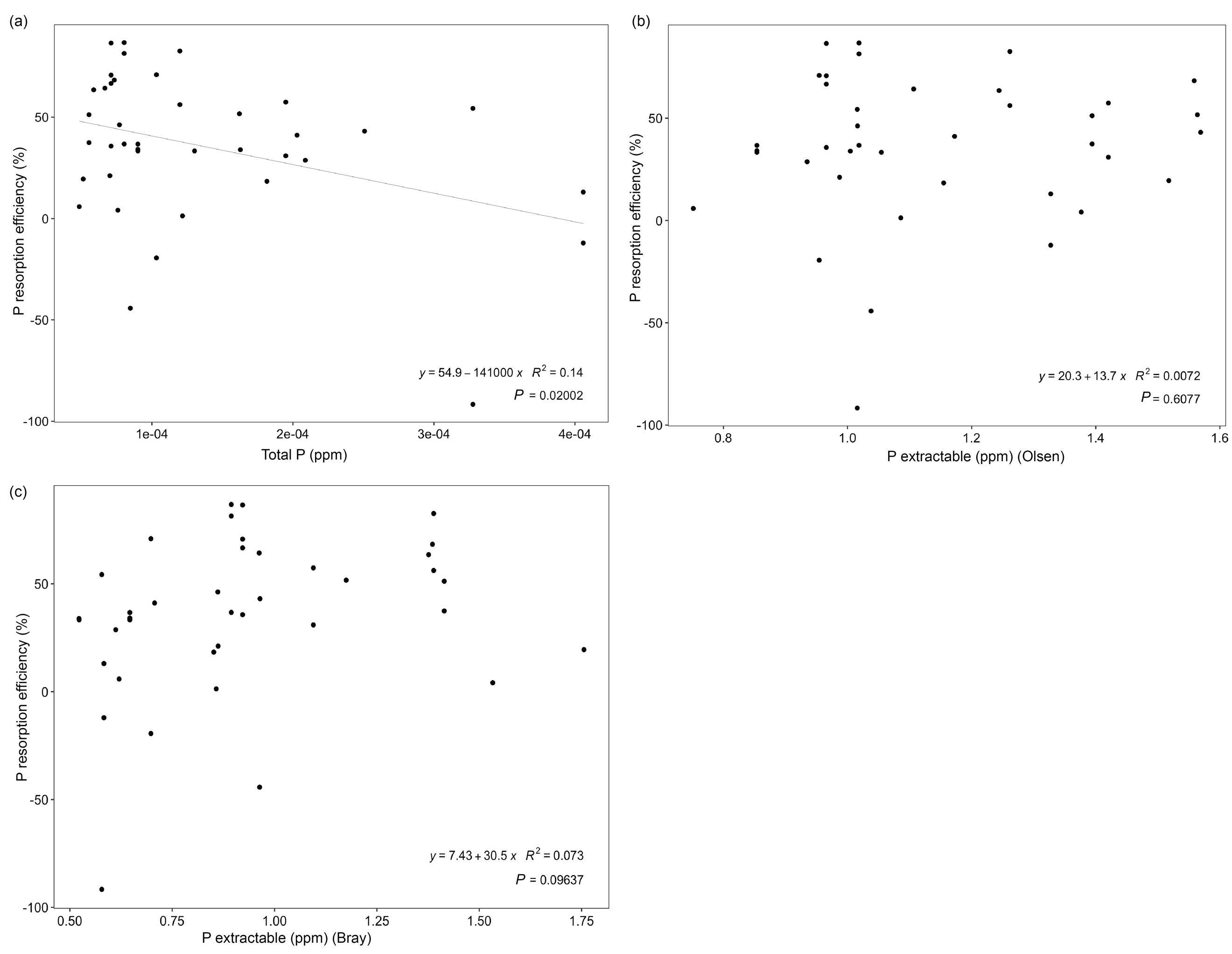
Figure S5. P resorption efficiency and its correlation with soil P (ppm) based on the 39 tropical tree species sampled at both study sites in the dry season. (a) P resorption efficiency versus total soil P, (b) P resorption efficiency versus soil extractable P (Olsen method) and (c) P resorption efficiency versus soil extractable P (Bray method). Coefficients for the significant regressions and *R*^2^ are displayed in the lower-right corner of the panel.

Figure S6. N and K resorption efficiencies and their correlations with soil N and K (dw/dw) based on the 39 tropical tree species. (a) N resorption efficiency versus soil N and (b) K resorption efficiency versus soil K.


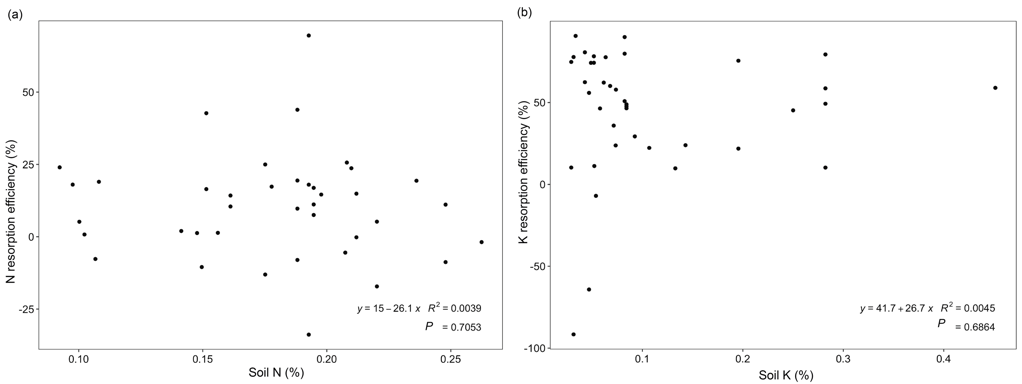


Figure S7. Nutrient resorption efficiency and its relationship with some functional traits based on the 39 tropical tree species. Significant relationships of (a) P resorption versus wood density, (b) K resorption versus wood density, (c) N resorption versus DBH and (d) P resorption versus SLA. Coefficients for the significant regressions and *R*^2^ are displayed in the lower-right corner of each panel.


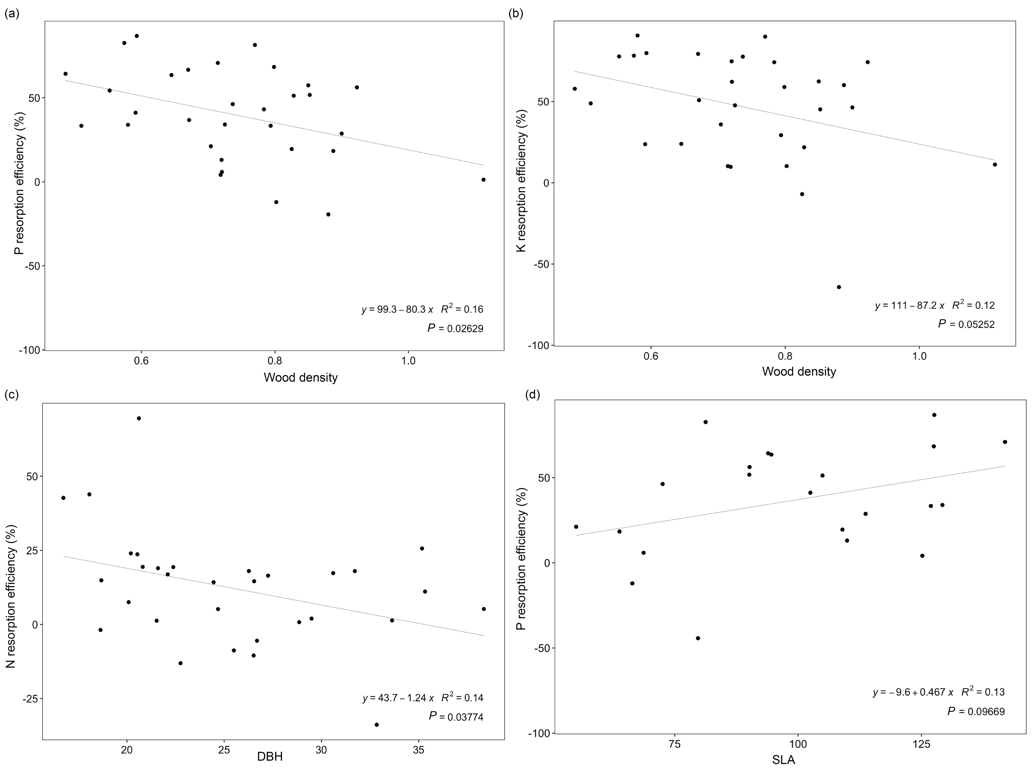


Deduction of the allometric coefficient (C) for estimating leaf weight based on DBH from a global data set published by Chave et al. (2014) which includes 2013 tree species (180 in Africa, 942 in the Neotropics and 891 in tropical Asia) at 31 tropical rainforest sites. Dry forests and woodlands were excluded to minimize bias towards deciduous trees.

Log (leaf weight) = log (basal area) + C

Basal area = π × (DBH/2)^2^.

The final model to infer leaf weight from basal area is:

Leaf weight = exp (log (basal area) + C')

where C'= C + RSE^2/2^ (Baskerville correction is needed here, because a Gaussian error in the log-transformed model is no longer Gaussian when exponential; the error becomes log-normal with a mean of RSE^2/2^, hence the correction).

The model fit is: C = -4.2569 and Residual standard error (RSE) =0.8704, such that:

C' = exp (-4.0153 + 0.8704^(2/2)^) = *0.02634434*

The resulting allometric equation is:

*Leaf weight* = 0.02634434 × basal area

where the basal area of a tree is π × (DBH/2)^2^.
